# Exceeding 30% Efficiency of Red Perovskite Quantum Dot Light-Emitting Diodes via Interparticle Energy Dissipation Suppression

**DOI:** 10.1007/s40820-026-02156-1

**Published:** 2026-03-19

**Authors:** Zhiwei Yao, Changsheng Liang, Chenghao Bi, Wenyuan Zhou, Ke Ren, Ming Deng, Shuo Ding, Chaoyu Xiang

**Affiliations:** 1 Laboratory of Advanced Nano-Optoelectronic Materials and Devices, Ningbo Institute of Materials Technology and Engineering, Chinese Academy of Science, Ningbo, 315201 People’s Republic of China; 2 College of Physics and Optoelectronic Engineering, Harbin Engineering University, Harbin, 150001 People’s Republic of China; 3 University of Chinese Academy of Sciences, No.1 Yanqihu East Road, Huairou District, Beijing, 101408 People’s Republic of China; 4 Laboratory of Advanced Nano-Optoelectronic Materials and Devices, Qianwan Institute of CNITECH, Ningbo, 315300 People’s Republic of China

**Keywords:** Self-repulsion ligands, Suppressed energy dissipation, Negligible redshift in Film, Red CsPbI_3_ quantum dots, Light-emitting diodes

## Abstract

The performance limits of perovskite quantum dot (QD) light-emitting diodes (PeLEDs) remain incompletely explored currently. Energy dissipation arising from interdot electronic coupling in QD solid films is readily induced by reduced interparticle distances and delocalized electron wavefunctions, thereby triggering severe nonradiative recombination and impeding further efficiency gains. To mitigate this energy loss, 1H,1H-undecafluorohexylamine (11-PFHA), characterized by pronounced steric self-repulsion and concentrated electron density distribution, was employed. The steric self-repulsion of 11-PFHA enlarges interdot spacing, while its concentrated electron distribution restructures the QDs’ surface electron distribution and establishes a higher interfacial electronic potential barrier between adjacent QDs. The treatment of 11-PFHA effectively suppresses electronic coupling and concomitant energy dissipation. Consequently, 11-PFHA-treated red-emitting perovskite QDs exhibit a near-unity photoluminescence quantum yield. PeLEDs fabricated with these optimized QDs achieve record external quantum efficiencies (EQEs), reaching 28.9% at 640 nm and 32.0% at 657 nm, indicating the highest EQE values reported to date.

**Supplementary Information:**

The online version contains supplementary material available at 10.1007/s40820-026-02156-1.

## Highlights

- A new quantum dot (QD) films with negligible redshift of photoluminescence spectra after film fabrication from QD solution.
- An electron barrier around QDs realized by adopting ligands with concentrated electron distribution.
- Record external quantum efficiency of light-emitting diodes based on CsPbI_3_ QDs (28.9% at 640 nm and 32.0% at 657 nm).

**Supplementary Information:**

The online version contains supplementary material available at 10.1007/s40820-026-02156-1.

## Graphical abstract

**Figure:**
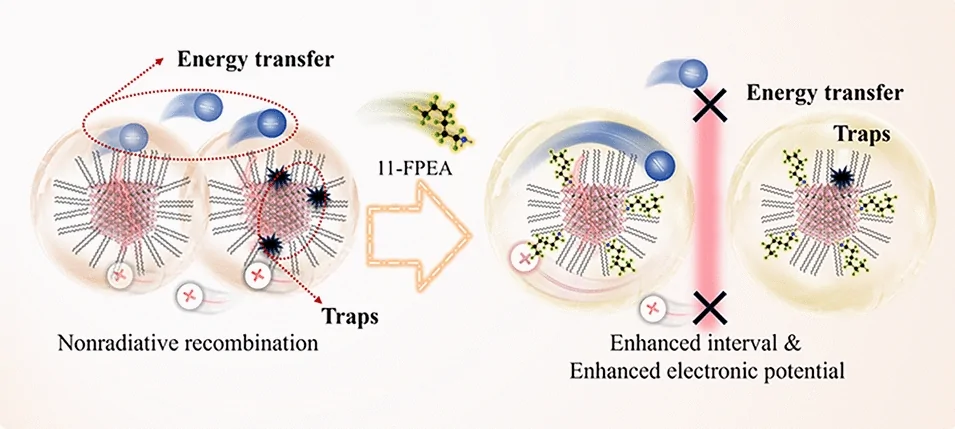

## Introduction

Metal halide perovskites (MHPs) have emerged as up-and-coming candidates for the next generation of displays attributed to their remarkable performance, and the distinctive optical characteristics of perovskite quantum dots (QDs), including widely tunable emission [1–3], high defect tolerance [4], high color purity [3, 5–7], high photoluminescence quantum yields (PLQY) [8], and the convenience of solution-process fabrication [2, 3, 9], have stimulated significant research interest, driving rapid advancement of this field [10–12]. However, to satisfy the color coordinate requirements for red primaries in displays, the intrinsic bandgap of CsPbI_3_ (∼700 nm emission) necessitates spectral tuning to shorter wavelengths [1, 4, 5, 13, 14]. Current approaches to achieve red emission comprise mixed-halide compositions (Br/I) or size-confinement in CsPbI_3_ QDs [15, 16]. Relative to mixed-halide systems, small-sized QDs demonstrate superior stability by avoiding halide segregation phenomena, thereby endowing them with enhanced applicability for high-performance optoelectronic devices [8, 17–19].

**Fig. 1:**
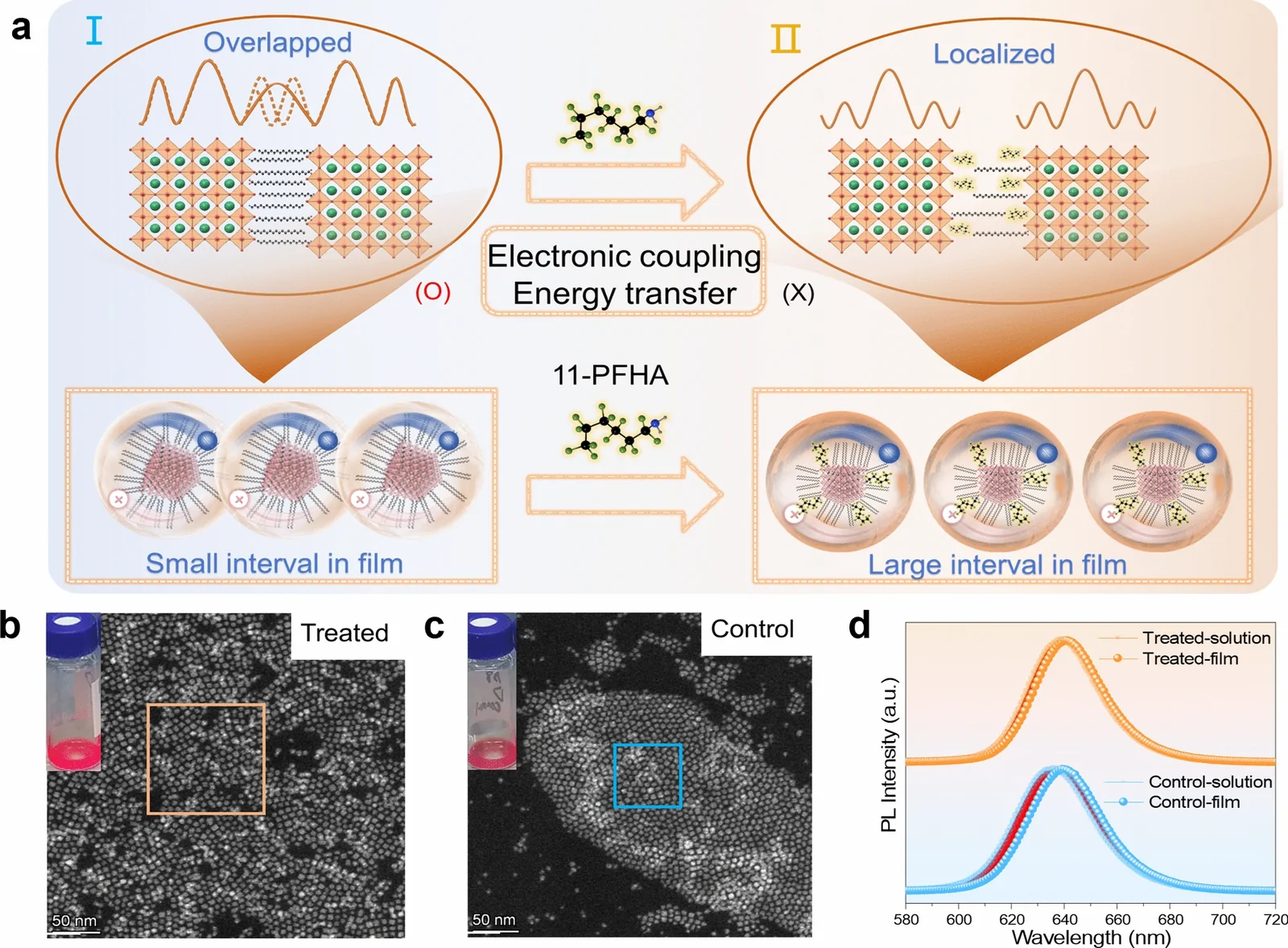
**a** Schematic illustration of the radiative processes of QDs before and after treatment. **b** TEM image of the treated sample. **c** TEM image of the control sample. **d** PL emission spectra of the control and treated QDs (640 nm) in both solution and film states

The performance of perovskite QDs is critically governed by the surface ligands attributable to the absence of a core-shell architecture. This dependence is exacerbated in small-sized QDs, where the surface-to-volume ratio is elevated, thereby increasing the susceptibility to ligand coverage. Consequently, augmented defect exposure occurs, promoting QD aggregation and the degradation of optical properties. Additionally, diminished interparticle spacing enhances electron cloud overlap between adjacent QDs, facilitating electronic coupling as demonstrated in Fig. 1a(I). Such intensified coupling may promote interdot energy transfer [5, 20, 21]. Correspondingly, energy dissipation associated with excitons coupling to charge carriers or defects is amplified [22–26]. As a result, the performance of the perovskite QD film and the related devices would also be compromised. Therefore, regulating electron distribution to suppress energy dissipation arising from electronic coupling constitutes a critical strategy for enhancing the performance of QD films and related devices [27].

To reduce the energy dissipation, the key point lies in minimizing the overlap of electron distribution between the adjacent QDs [27]. The widely adopted strategy focuses on spatial segregation of QDs via the implementation of long-chain ligands [28] or insulating shells like zeolite [29]. However, these approaches significantly impair charge transport conductivity within fabricated films. Consequently, while treated QDs exhibit enhanced environmental stability and colloidal dispersibility, their utility in full-color active light-emitting devices remains unrealized [28, 29]. Other strategies employing conjugated organic materials facilitate QD isolation into monodisperse phases [27]. Unfortunately, such methodologies require precise QD-organic conductors energy level alignment, coupled with precise exciton recombination interface management between QDs and conductive organic frameworks.

In this work, 1H,1H-Undecafluorohexylamine (11-PFHA) was adopted to partially replace oleylamine (OAm), restructuring electron distribution around the QD surface to suppress electron coupling-related energy dissipation. The self-repellent molecular architecture, attributed to C–F bond interactions, introduced steric hindrance between adjacent QDs, thereby expanding interparticle spacing and forming a spatially decoupled network as presented in Fig. 1a(II). Moreover, concentrated electron distribution at the 11-PFHA fluorocarbon group was demonstrated to establish an electronic potential barrier, enhancing quantum confinement within QDs while suppressing interparticle energy transfer. Consequently, photoluminescence (PL) spectra of treated QDs exhibited a negligible spectral shift upon film assembly. The PLQY was elevated from 91% to nearly unity in the treated QDs. When incorporated into perovskite light-emitting diodes (PeLEDs), the devices based on the treated QDs achieved an external quantum efficiency (EQE) as high as 28.9% at 640 nm and 32.0% at 657 nm, significantly surpassing that of the control device, representing the highest value reported to date. The operational lifetimes also demonstrate substantial improvement by 2.5 and 10 times for devices based on treated QD emission at 640 and 657 nm, respectively.

## Experimental Section

### Materials

Cesium carbonate (Cs_2_CO_3_, Sigma-Aldrich, 99.999%), oleic acid (OA, Alfa Aesar, 90%), oleylamine (OAm, Aladdin, 90%), 1-octadecene (ODE, Alfa Aesar, 90%), lead (II) iodide (PbI_2_, Advanced Election Technology Co., Ltd., 99.9%), zinc iodide (ZnI_2_, Aladdin, 99.999%), methyl acetate (Macklin, 98%), octane (Aladdin, > 99%), ITO Advanced Election Technology Co., Ltd., about 7 Ohm sq^−1^), PEDOT: PSS (4083, Xi’an Bath Sunlight Technology Co., Ltd), PTAA (Linkzill), TmPyPB (Xi’an Bath Sunlight Technology Co., Ltd), PO-T2T (Xi’an Bath Sunlight Technology Co., Ltd), LiF (Xi’an Bath Sunlight Technology Co., Ltd), and 1H,1H-Undecafluorohexylamine (11-PHFA, TCI, 97.0%).

### Preparation of QDs

#### Synthesis of Cs-OA

Typically, 100 mg of Cs_2_CO_3_, 10 mL ODE and 0.7 mL of OA were added into a three-neck flask (25 mL), the solution will be degassed at the room temperature, 60, and 120 °C for 30 min and filled with Ar, respectively, and to ensure the inert atmosphere, after that, the solution should be bubbled at 120 °C for 10 min before being used.

#### Preparation of CsPbI_3_ QDs

 173 mg of PbI_2_ and 10mL of ODE were added to the three-neck flask. The solution will be bubbled at 120 °C for 30 min to get rid of water and oxygen and form an inert atmosphere. Then, 3 mL OA and 3 mL OAm were injected to form a transparent solution. The mixture will be bubbled for 15 min to get rid of the extra water and oxygen brought by OA and OAm. Then, the temperature of the solution was raised to 170 °C quickly, and 1.6 mL of Cs-OA was injected into the precursor as soon as possible. The solution would turn red immediately, the raw solution will be cooled with ice water after 5 s to complete the synthesis.

The crude solution was transferred to a centrifuge tube and subjected to an initial centrifugation at 10,000 rpm for 1 min to remove large aggregates. The resulting supernatant was then collected and mixed with 30 mL of methyl acetate, followed by centrifugation at 7000 rpm for 3 min. The precipitate from this step, which comprised small-sized QDs with an emission peak at 657 nm, was collected. The corresponding supernatant was retained. Next, 45 mL of methyl acetate was added to this supernatant, and the mixture was centrifuged at 10,000 rpm for 3 min. The precipitate obtained (emission at 640 nm) was collected. Finally, all collected precipitates were redispersed in n-octane to form a 10 mg mL^−1^ stock solution for device fabrication.

Treatment of 11-PFHA QDs: 100 μL of QD solution was added into a bottle (2 mL), then 5 μL of 11-PFHA was added into the solution in the glovebox, and the solution was stirred overnight for the next process.

#### Fabrication of PeLEDs

Firstly, the ITO-deposited glass was washed with deionized water, acetone, and ethyl alcohol in the ultrasonic machine. The ITO glass was treated with Ultraviolet ozone (UVO) for 30 min, and PEDOT:PSS was spin-coated at 6000 rpm for 30 s, followed by baking at 150 °C for 10 min in a fume hood. Then the substrates were transferred into the glovebox filled with an inert atmosphere, PTAA was spin-coated at 1000 rpm, 30 s, and baked at 130 °C for 30 min. After the deposition of PTAA, QDs were spin-coated at 6000 rpm for 30 s to form the emitting layer (EML). After the EML, the substrates were transferred to the evaporation device for the deposition of TmPyPB, PO-T2T, LiF, and Al. Then, the devices were encapsulated with cover glass before being tested.

### Characterizations

UV–vis absorption spectra were obtained by PerkinElmer Lambda 950. Photoluminescence spectra were obtained by Horiba FL3-111 with an excitation source at 400 nm, and photoluminescence excitation profiles were obtained by Horiba FL3-111 with the emission peak at 640 and 657 nm. Transmission electron microscopy (TEM) pictures were captured with Talos F200X. For sample preparation, the as-prepared QD stock solutions (directly applicable for device fabrication), both control and 11-PFHA-treated QDs, were first diluted to the same extent with* n*-hexane. Subsequently, the diluted solutions were drop-cast onto copper grids, followed by TEM analysis. The PL Decay profiles were detected and collected by using an Edinburgh Instruments spectrometer (FLS1000). The FT-IR details were collected by a Fourier-transform infrared spectrometer (Thermelfeld, IS50). X-ray photoelectron spectroscopy (XPS) results were captured by an ESCALAB 250Xi. Ultraviolet photoelectron spectroscopy (UPS) was carried out by an ESCALAB 250Xi. The PLQY results were obtained by FL3-111. The transient absorption (TA) decay spectra were measured using a 1030 nm femtosecond laser as the probing light source, which was generated through an optical parametric amplifier (OPA) to obtain a 400 nm excitation light, and using an Ultrafast Transient Absorption Spectrometer (TIME-TECH SPECTRA, TA-ONE-1) as the detector. The light outcoupling efficiency of perovskite light-emitting diodes (PeLEDs) was calculated using the open-source oledpy package (GitHub repository: https://github.com/jsbangsund/oledpy, Copyright © 2019 John Bangsund, licensed under MIT License). This package is built on a classical dipole emission model and has been validated against commercial Setfos software and literature data, with absolute outcoupling efficiency values agreeing within 1%–2% and spectral trends (e.g., with layer thickness or refractive index) fully reproducible. All simulations were performed for a bottom-emitting geometry (light emission through the substrate), consistent with the current capability of oledpy. The electroluminescence performance of devices was measured with the same setup reported before [7, 8, 30]. Electroluminescence spectra were obtained using an Ocean Optics USB 2000 + spectrometer with the devices driven at a constant current with a Keithley 2400 source meter. The J–L–V characteristics of the devices were taken under ambient conditions with a Keithley 2400 source meter measuring the sweeping voltages and currents, and a Keithley 6485 Picoammeter, together with a calibrated silicon detector (Edmund) measuring light intensities. Luminance was calibrated using a photometer (Luminance meter LS-160) with the assumption of the Lambertian emission pattern of all devices. The operational lifetime test was conducted under ambient conditions at room temperature (22 ± 2 °C) using a commercialized lifetime test system (Guangzhou Jinghe Equipment Co., Ltd). The devices were encapsulated with Nagase UV epoxy resin XNR5516Z(C)-SA1 and capping glasses. After obtaining the current, voltage, and EQE through testing, the current efficiency is calculated from the EQE based on the collected spectra. Subsequently, the final luminance was derived from the device current. These calculations were currently performed by using preprogrammed software. 

## Results and Discussion

### Mechanism of 11-PFHA Treatment

The QDs were synthesized as follows, with some modifications [31]. Fourier-transform infrared (FT-IR) measurements were conducted to verify the implementation of this surface treatment, and the results are shown in Fig. S1. The stretching vibration absorption peak of the C–F bonds was detected and highlighted with a dotted line at 1240 cm^−1^, confirming the presence of 11-PFHA in the treated sample. Complementary X-ray photoelectron spectroscopy (XPS) measurements were carried out, and the results are shown in Fig. S2. The binding energy peaks of Pb 4*f* shifted from 138.05 and 142.90 eV to 138.20 and 143.05 eV, respectively, indicating bonding of Pb to a high-electronegativity element such as fluorine [2, 32, 33]. Besides, QDs treated by different amount of 11-PFHA were conducted and the optical performance are shown in Fig. S3. The concentration of 5 μL (100 μL QDs) are adopted as the optimal dose.

Transmission electron microscopy (TEM) measurements were carried out to verify the self-repulsion of QDs with the assistance of 11-PFHA, and the results are presented in Fig. 1b. The treated QDs exhibited improved dispersion, whereas in the control sample (Fig. 1c), the QDs were aggregated on the scale of hundreds of nanometers. This difference confirms that 11-PFHA addition enhances interparticle spacing as designed. Statistical analysis of interparticle spacing for control and treated QDs is presented in Fig. S4. The average spacing of treated QDs is measured as 4 nm, exceeding the control sample (2 nm), indicating enhanced dispersion. Besides, as the pictures shown in the insets of Fig. 1b, c, treated QD solution displays higher optical transparency, indicating the presence of large-scale aggregates, likely in the range of several hundred nanometers, within the control sample [34, 35]. The inset solution images in Fig. 1b, c further demonstrate superior QD separation, with the treated system displaying increased transparency and luminosity [36] relative to the control under ambient illumination.

Ultraviolet–visible absorption (UV–vis), photoluminescence (PL) and PL excitation (PLE) spectroscopy were performed to characterize the optical properties of control and 11-PFHA-treated QD samples. As demonstrated in Fig. S5a, b, the absorption spectra remained unchanged following 11-PFHA treatment, signifying unaltered QD phase and core dimensions. The PLE curves of the samples before and after treatment display an almost identical trend to the absorption. Additionally, the PLE intensity in the short-wavelength region exhibits a slight enhancement (highlighted with a red circle), indicating that the luminescent efficiency of QDs under high-energy light has been improved. This implies that energy dissipation between QDs within this system has been effectively suppressed. Contemporaneously, PL (Figs. 1d and S6) details revealed profound interdot differences upon integration within films. Control QDs displayed substantial redshifting post-film fabrication relative to solution-state spectra. Conversely, treated QDs manifested exceptional spectral stability (A minimal film–solution displacement was observed in QD emission at 640 nm, whereas the PL peak exhibited complete overlap in QD emission at 657nm). This suppression of solution-to-film PL peak displacement elucidates significantly attenuated interparticle electronic coupling within the EML based on 11-PFHA-treated QDs [5, 27, 37].

### Characterization of Different QDs and Films

**Fig. 2:**
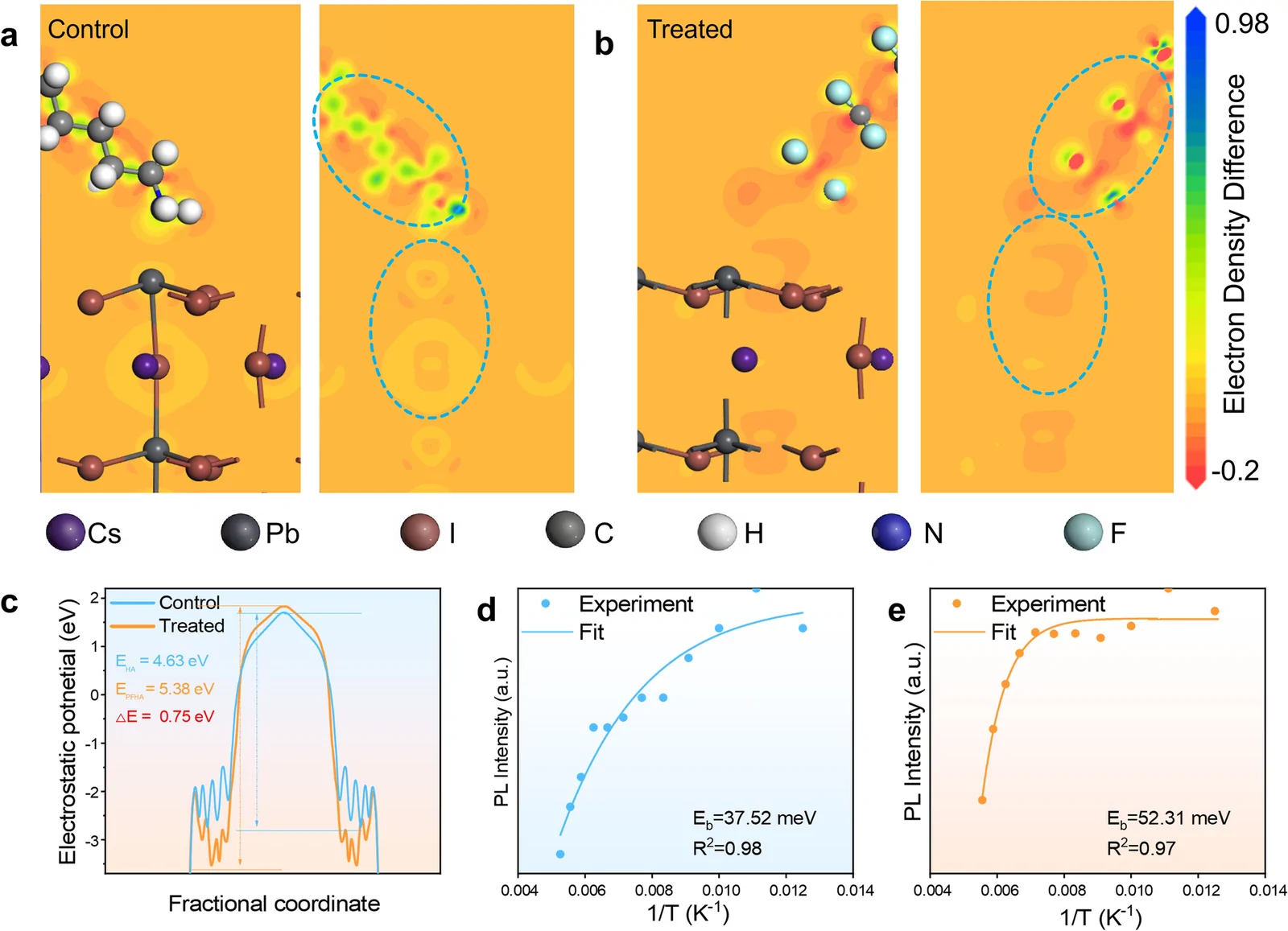
Schematic diagram of electron density difference between QDs passivated with **a** alkylamine and** b** 11-PFHA. **c** Electrostatic potential along the ligand between adjacent QDs. The integrated PL intensity of the **d** control QDs and **e** treated QDs as a function of reciprocal temperature

To elucidate the role of 11-PFHA in QD optimization, density functional theory (DFT) calculations were performed on alkylamine-passivated versus 11-PFHA-treated QDs (structural models are shown in Fig. S7). Electron density distributions across ligand interfaces were mapped (Figs. 2a and S8), revealing pronounced fluorine-induced charge polarization. The high electronegativity of fluorine atoms in 11-PFHA promotes significant electron density localization around C–F bonds, establishing strong molecular dipoles that generate intermolecular electrostatic repulsion. Consequently, 11-PFHA passivation induces electron density redistribution toward surface ligands, reconstituting QD surface electronic landscapes and establishing electron potential barriers. This charge confinement also spatializes excitons within QD cores, thereby enhancing excitonic confinement and improving optoelectronic efficacy.

Electronic potential profiles along inter-QD ligand pathways were simulated (Fig. 2c), demonstrating substantial differences between 11-PFHA-treated QDs and the control samples (QDs passivated with alkylamine). Alkylamine-passivated QDs exhibited a potential well of − 2.74 eV and a peak of 1.69 eV, yielding a 4.63 eV electronic potential barrier (Fig. S9). In contrast, 11-PFHA passivation produced a deeper well (− 3.56 eV) and elevated peak (1.82 eV), resulting in a higher electronic potential barrier of 5.38 eV, a 0.75 eV enhancement relative to the alkylamine-treated one. This heightened barrier impedes interdot energy transfer, suppressing electronic coupling between adjacent QDs. Consequently, nonradiative processes related to interdot energy dissipation are suppressed in 11-PFHA-treated QDs.

Temperature-dependent photoluminescence and time-resolved photoluminescence (TRPL) decay spectroscopy were performed to elucidate the enhancement of the optical performance in 11-PFHA-passivated QDs relative to alkylamine-passivatedQDs. After 11-PFHA, a ligand with a low dielectric constant, was introduced into the QD solution, the confinement of QDs was effectively enhanced under the strong electronegativity of fluorine atoms and the resulting dielectric effect. Exciton binding energies (E_b_) were quantitatively determined from Eq. (1) (fittings are shown in Fig. 2d, e), yielding values of 37.52 and 52.31 meV for the respective control and 11-PFHA-passivated samples. The E_b_ in the treated sample was improved by the obvious dielectric mismatch between QDs and ligands [2, 38], further inhibiting the dissociation of excitons in 11-PFHA-treated QDs. Besides, the intensified exciton confinement suppresses nonradiative recombination pathways, diminishing energy dissipation losses [39–41]. TRPL kinetics (Fig. S10) of 11-PFHA-passivated QDs manifest substantially attenuated nonradiative decay channels relative to control QDs, with the average lifetime enhanced from 10.15 to 13.62 ns (Table S1), suggesting that the treated QDs with superior radiative efficiency [42]. The enhanced PLQY of the solution and the slight decrease of film also indicated the enhanced performance and suppressed interparticle dissipation in the treated sample (Fig. S11).

1 $$I\left(t\right)=\frac{{I}_{0}}{1+A\mathrm{exp}\left(\frac{{E}_{b}}{{k}_{B}T}\right)}$$

**Fig. 3:**
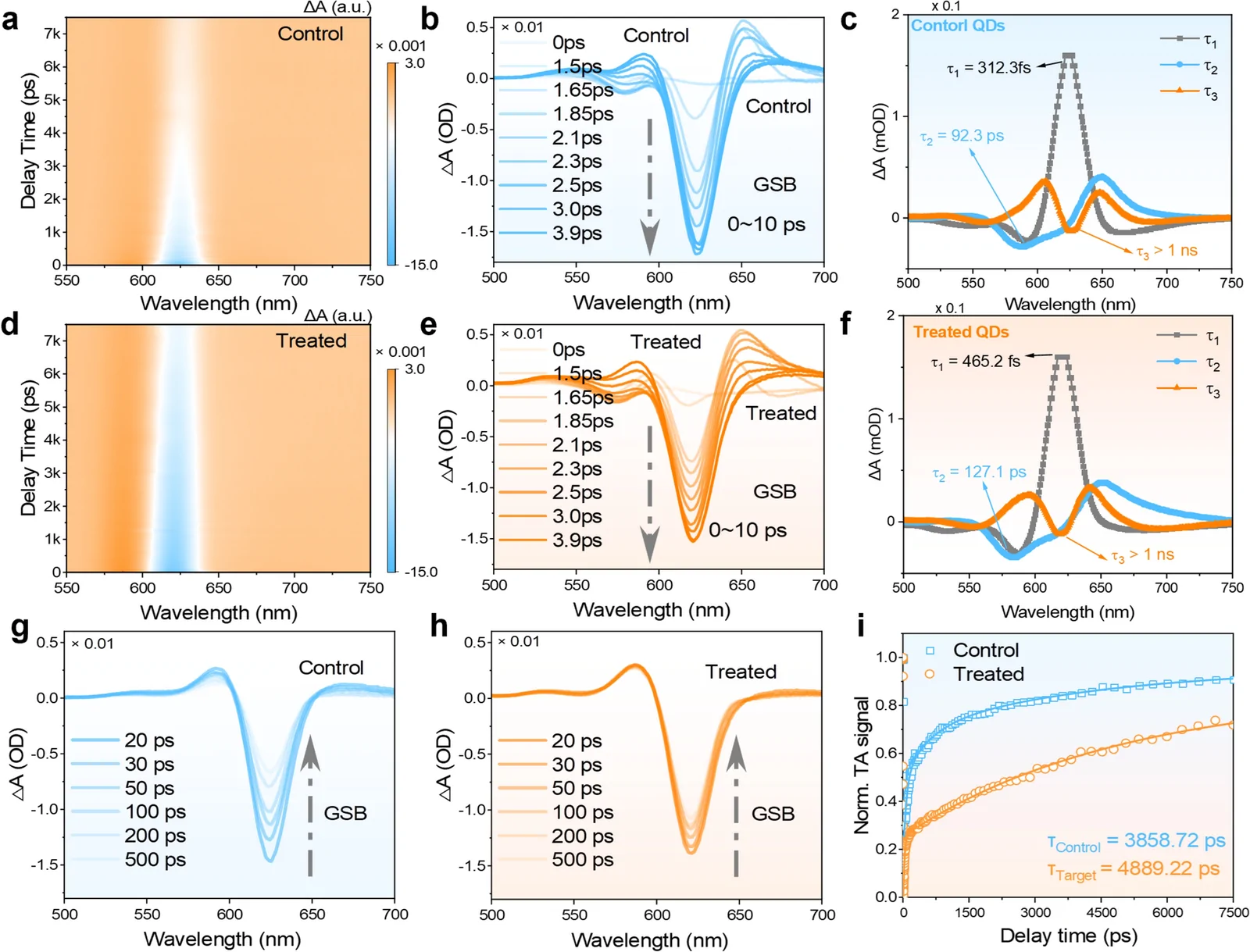
2D pseudo-color fs-TA plots of **a** control QDs and **d** optimized QDs. The fs-TA spectra of the establishment process for **b** the control QDs and **e** the treated QDs within 10 ps. Decay-associated spectra for **c** control and **f** treated QDs. The fs-TA spectra at different delay times within 500 ps of **g** control QDs and **h** treated QDs. **i** TA spectra comparisons of GSB peaks recovery dynamics of the control QDs and the treated QDs within 6000 ps

Time-resolved transient absorption (TA) spectroscopy was conducted on control and 11-PFHA-passivated QD samples, with the results detailed in Fig. 3. Two-dimensional pseudo-color fs-TA spectra (Fig. 3a, d) and the TA spectra at various delay times within 500 and 10 ps of the control and treated samples are also explored. Both the control and treated QDs demonstrate comparable ground-state bleaching (GSB) at ~ 620 nm in both systems, confirming an identical bandgap. Nevertheless, the treated QDs exhibit significantly prolonged GSB recovery kinetics relative to the control, indicating substantial suppression of the fast recombination channels [43].

The early-stage spectra within 10 ps post-excitation (Fig. 3b, e) and the global fitting (Fig. 3c, f) of the TA results were employed to reveal the characteristic of decay-associated spectra (DAS). As shown in Fig. 3c, the related characteristic time constants are τ_1_ = 312.3 fs, τ_2_ = 92.3 ps and τ_3_ > 1ns for the control sample, while that of the treated QDs are τ_1_ = 465.2 fs, τ_2_ = 127.1 ps and τ_3_ > 1ns (Fig. 3f). The three decay parameters (τ_1_, τ_2_, τ_3_) account for the processes of hot exciton relaxation, exciton trapping to the trap states at band edge, and exciton recombination, respectively [44, 45]. The former two processes in the treated sample are slower than the control one, revealing that the hot exciton relaxation and trap-assisted processes are restrained after being treated with 11-PFHA. In the treated QDs, the suppression of trap-assisted recombination should be attributed to the passivation of 11-PFHA, the prolonged hot carrier cooling process can be explained as the weakened charge-phonon coupling which caused by the increased distance between QDs and the suppressed lattice vibrations brought by the self-repulsion and rigidity of ligands [46]. In addition, the enhanced screening and Coulomb interaction of the QDs brought by 11-PFHA tend to localize charge carriers within the perovskite QDs, reduce energy transfer between particles, and slow down the cooling rate of hot carriers [47, 48]. The dynamics of the GSB establishment process for control and treated QDs were carried out (Fig. S12), and the lifetimes are 480 and 840 fs of the control and treated samples, implying the enhanced localization of charge and reduced interaction between adjacent QDs. Besides, normalized GSB recovery dynamic spectra for all samples are shown in Fig. 3i. The control sample exhibits an average lifetime of 3858.72 ns, while that of the treated one is 4889.22 ns, longer than the control one, confirming the reduced nonradiative recombination.

### Device Performance

**Fig. 4:**
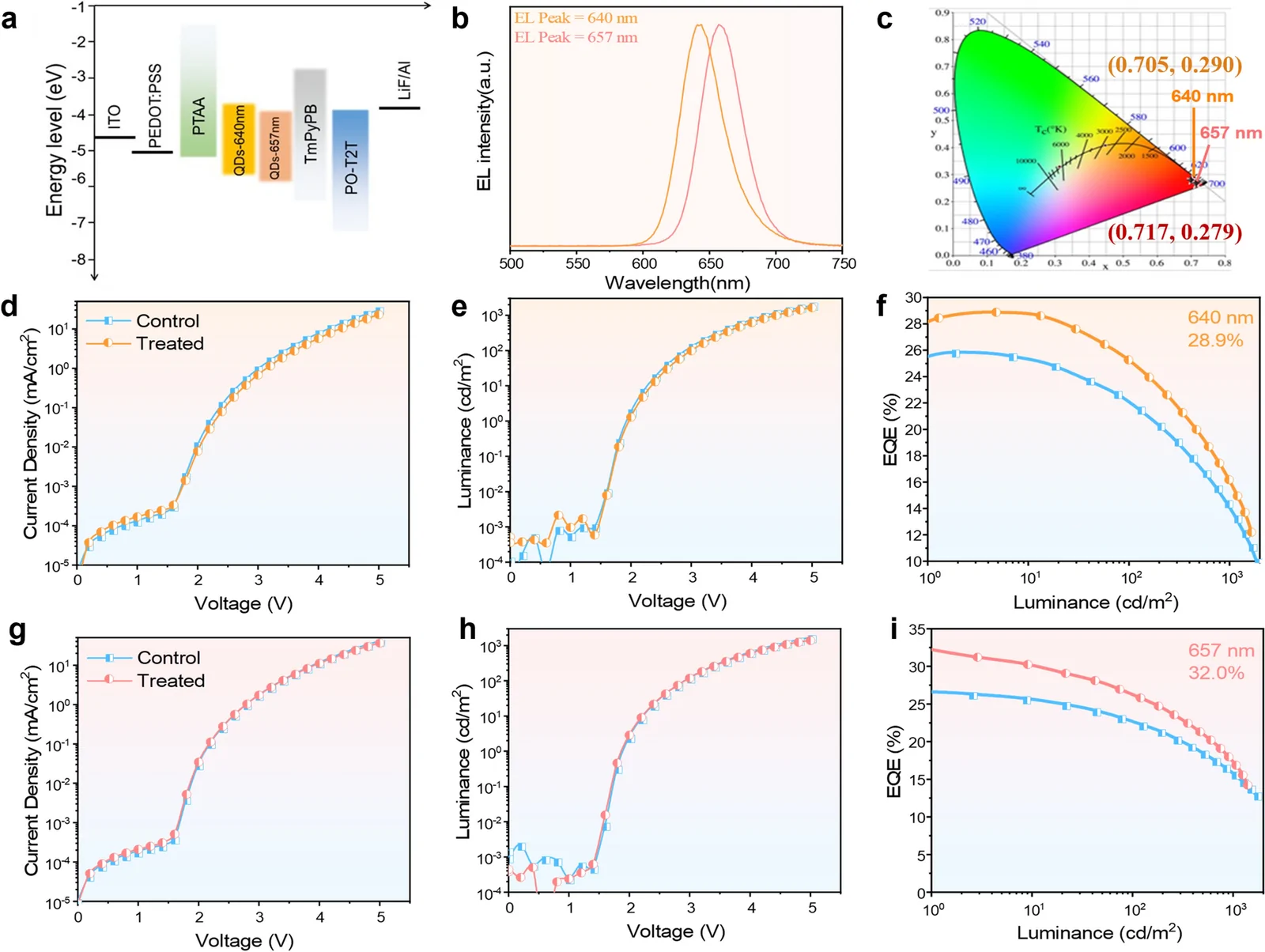
**a** Diagram of the energy level of the device. **b** EL spectra of PeLEDs. **c** CIE picture of the red PeLEDs with the emission of 640 and 657 nm. **d** Current density-voltage results, **e** luminance-voltage results, **f** EQE-luminance results of PeLEDs based on the control and the optimized QDs with the emission of 640 nm. **g** Current density-voltage results, **h** luminance-voltage results, **i** EQE-luminance results of PeLEDs based on the control and the optimized QDs with the emission of 657 nm

To evaluate the electroluminescent properties of perovskite QDs treated with 11-PFHA, PeLEDs were fabricated based on QDs with varying sizes, emitting at 640 and 657 nm. The TEM picture and the statistics of the size of QD emission at 640 and 657 nm are shown in Fig. S13. The average size of QD emission at 640 nm is about 4.75 nm, and that of QD emission at 657 nm is about 6.25 nm. Control and treated QDs were integrated into devices with the architecture ITO/PEDOT: PSS/PTAA/QDs/TmPyPB/PO-T2T/LiF/Al, with the cross-sectional TEM images shown in Fig. S14. Ultraviolet photoelectron spectroscopy (UPS) was employed to determine the energy landscapes of different QDs (Fig. S15). As shown by the results, the HOMO level of the 640 nm-emitting sample shifts from − 5.71 to − 5.53 eV upon treatment, whereas that of the 657 nm-emitting sample shifts from − 5.81 to − 5.63 eV after treatment, promoting the hole injection as shown in Fig. S16. Notably, the HOMO levels of treated QDs are universally elevated and exhibit improved alignment with that of PTAA, which facilitates the attainment of high external quantum efficiency (EQE). The energy band alignment of the red PeLEDs (emission at 640 and 657 nm) is illustrated in Figs. 4a and S15.

As demonstrated by the electroluminescence (EL) spectra (Fig. 4b) and the CIE chromaticity coordinates (Fig. 4c), devices incorporating treated QDs exhibited a saturated red emission. Current density–voltage (J–V), luminance–voltage (L–V), and external quantum efficiency–current density (EQE–J) characteristics of PeLEDs based on control and treated QDs (emission at 640 nm) are shown in Fig. 4d–f, while those emitting at 657 nm are presented in Fig. 4g–i. Due to the intrinsically insulating nature of 11-PFHA and the consequent reduction in electrical conductivity of the QDs, devices utilizing treated QDs exhibited a marginally decreased current density (Fig. 4d, g), alongside a corresponding slight reduction in luminance (Fig. 4e, h). Nevertheless, the EQE of treated QD-based devices demonstrated notable enhancement along with the increasing current density (Fig. 4f, i), which should be attributed to suppressed energy dissipation. Besides, the current efficiency (CE) of devices based on treated QDs enhanced from 7.8% and 16.6% to 9.4 and 18.6 cd A^-1^, presenting the same trend as EQE (Fig. S18). PeLEDs based on treated QDs achieved peak EQE values of 28.9% (640 nm) and 32.0% (657 nm) at 1 cd m^−2^, with average EQEs of 26% and 29% (Fig. S19), demonstrating good reproducibility and setting a new record for red PeLEDs with conventional architecture (Fig. S20) of PeLEDs based on CsPbI_3_ QDs from 620 to 680 nm.

Furthermore, PL spectra at different bias of devices based on different QDs and the PL spectra before and after T_50_ measurement of devices based on different QDs are captured and the result are shown in Fig. S21. The EL peak of the devices at different bias based on different QDs kept unchanged, implying a stable emission and promising performance of the synthesized QDs (Fig. S21a, b). The spectra of devices before and after T_50_ measurement also shown in Fig. S21c, the broadening and tailing of the EL spectrum are relatively minor, illustrating that the operational stability of the treated QDs were improved. Furthermore, we have also performed optical simulations and angular-dependent EL intensity measurements (Figs. S22 and S23). The optical simulation results also confirm the credibility of the efficiency of our devices (Fig. S22) with the thickness as shown in the STEM picture. Besides, the device exhibits a favorable Lambertian emission profile (Fig. S23). Operational stability measurements were conducted at approximately 100 cd/m^-2^, and the results revealed significant improvements, with treated QD-based devices exhibiting 2.5-fold (640 nm) and 20-fold (657 nm) enhancements in operational lifetime, as indicated by the decay profiles in Figs. S24 and S25, indicating that 11-PFHA can enhance both the efficiency and the operational stability.

## Conclusion

In summary, further improvements in the efficiency of high-performance red PeLEDs are impeded by energy dissipation resulting from reduced interparticle spacing and enhanced electron coupling. To address this dilemma, we introduce 11-PFHA, a ligand characterized by pronounced self-repulsion and concentrated electron density, to suppress energy dissipation by increasing the spacing between adjacent QDs and restructuring their electron distribution. Following 11-PFHA treatment, the PLQY of the QDs increased from 91% to near unity, while the average interparticle distance in the film expanded, effectively eliminating the redshift of films compared with the solution. DFT calculations indicate that the treated QDs exhibit an elevated electronic potential barrier along the ligand direction, enhancing quantum confinement and suppressing interparticle energy dissipation. Furthermore, TA spectroscopy revealed prolonged GSB formation and decay processes, corroborating the suppression of energy transfer processed and nonradiative losses. When integrated into PeLEDs, the 11-PFHA-treated QDs demonstrated significantly improved EQE compared to control devices. Notably, peak EQE values of 28.9% (640 nm) and 32.0% (657 nm) were obtained, the highest reported for red PeLEDs to date. This study elucidates a novel strategy for fabricating high-performance red PeLEDs, paving the way for advancements in full-color displays. By leveraging self-repulsive ligands to mitigate energy dissipation in QD films, we posit a scalable approach for further breakthroughs in PeLED efficiency.

## Supplementary Information

Below is the link to the electronic supplementary material.

Supplementary file1 (DOCX 6534 KB)

## References

[1] L. Kong, Y. Sun, B. Zhao, K. Ji, J. Feng et al., Fabrication of red-emitting perovskite LEDs by stabilizing their octahedral structure. Nature **631**(8019), 73–79 (2024). 10.1038/s41586-024-07531-9 38867044 10.1038/s41586-024-07531-9

[2] C. Bi, Z. Yao, J. Hu, X. Wang, M. Zhang et al., Suppressing auger recombination of perovskite quantum dots for efficient pure-blue-light-emitting diodes. ACS Energy Lett. **8**(1), 731–739 (2023). 10.1021/acsenergylett.2c02613

[3] C. Bi, Z. Yao, X. Sun, X. Wei, J. Wang et al., Perovskite quantum dots with ultralow trap density by acid etching-driven ligand exchange for high luminance and stable pure-blue light-emitting diodes. Adv. Mater. **33**(15), e2006722 (2021). 10.1002/adma.202006722 33629762 10.1002/adma.202006722

[4] H. Li, Y. Feng, M. Zhu, Y. Gao, C. Fan et al., Nanosurface-reconstructed perovskite for highly efficient and stable active-matrix light-emitting diode display. Nat. Nanotechnol. **19**(5), 638–645 (2024). 10.1038/s41565-024-01652-y 38649747 10.1038/s41565-024-01652-y

[5] Y.-K. Wang, H. Wan, S. Teale, L. Grater, F. Zhao et al., Long-range order enabled stability in quantum dot light-emitting diodes. Nature **629**(8012), 586–591 (2024). 10.1038/s41586-024-07363-7 38720080 10.1038/s41586-024-07363-7

[6] Z. Yao, C. Bi, R. Xu, X. Zhang, L. Qian et al., Decoupling the exciton-carrier interaction for highly efficient pure blue perovskite light-emitting diodes exceeding 20%. Adv. Mater. **37**(42), e20131 (2025). 10.1002/adma.202420131 40757913 10.1002/adma.202420131

[7] X. Zhang, Q. Wang, Z. Yao, M. Deng, J. Wang et al., Stable perovskite quantum dots light-emitting diodes with efficiency exceeding 24%. Adv. Sci. **10**(36), 2304696 (2023). 10.1002/advs.202304696 10.1002/advs.202304696 PMC10754115 37890450

[8] Y. Li, M. Deng, X. Zhang, T. Xu, X. Wang et al., Stable and efficient CsPbI_3_ quantum-dot light-emitting diodes with strong quantum confinement. Nat. Commun. **15**, 5696 (2024). 10.1038/s41467-024-50022-8 38972890 10.1038/s41467-024-50022-8 PMC11228028

[9] Z. Yao, C. Bi, A. Liu, M. Zhang, J. Tian, High brightness and stability pure-blue perovskite light-emitting diodes based on a novel structural quantum-dot film. Nano Energy **95**, 106974 (2022). 10.1016/j.nanoen.2022.106974

[10] C. Bi, X. Sun, X. Huang, S. Wang, J. Yuan et al., Stable CsPb_1–__*x*_Zn_*x*_I_3_ colloidal quantum dots with ultralow density of trap states for high-performance solar cells. Chem. Mater. **32**(14), 6105–6113 (2020). 10.1021/acs.chemmater.0c01750

[11] C. Guo, C. Bi, S. Wei, K. Ren, X. Huang et al., Highly efficient and stable CsPbI_3_ Perovskite quantum dots light-emitting diodes through synergistic effect of halide-rich modulation and lattice repair. Small **21**(8), 2409630 (2025). 10.1002/smll.202409630 10.1002/smll.202409630 39831832

[12] C. Bi, S.V. Kershaw, A.L. Rogach, J. Tian, Improved stability and photodetector performance of CsPbI_3_ Perovskite quantum dots by ligand exchange with aminoethanethiol. Adv. Funct. Mater. **29**(29), 1902446 (2019). 10.1002/adfm.201902446

[13] N. Wang, L. Cheng, R. Ge, S. Zhang, Y. Miao et al., Perovskite light-emitting diodes based on solution-processed self-organized multiple quantum wells. Nat. Photonics **10**(11), 699–704 (2016). 10.1038/nphoton.2016.185

[14] J. Jiang, M. Shi, Z. Xia, Y. Cheng, Z. Chu et al., Efficient pure-red perovskite light-emitting diodes with strong passivation *via* ultrasmall-sized molecules. Sci. Adv. **10**(18), eadn5683 (2024). 10.1126/sciadv.adn5683 38701203 10.1126/sciadv.adn5683 PMC11067999

[15] Y.-H. Song, B. Li, Z.-J. Wang, X.-L. Tai, G.-J. Ding et al., Intragrain 3D Perovskite heterostructure for high-performance pure-red Perovskite LEDs. Nature **641**(8062), 352–357 (2025). 10.1038/s41586-025-08867-6 40335712 10.1038/s41586-025-08867-6

[16] J. Zhang, B. Cai, X. Zhou, F. Yuan, C. Yin et al., Ligand-induced cation-π interactions enable high-efficiency, bright, and spectrally stable rec. 2020 pure-red perovskite light-emitting diodes. Adv. Mater. **35**(45), e2303938 (2023). 10.1002/adma.202303938 37464982 10.1002/adma.202303938

[17] J. Guo, M. Xie, H. Li, L. Zhang, L. Zhang et al., High efficiency and low roll-off pure-red Perovskite LED enabled by simultaneously inhibiting Auger and trap recombination of quantum dots. Nano Lett. **24**(21), 6410–6416 (2024). 10.1021/acs.nanolett.4c01441 38767286 10.1021/acs.nanolett.4c01441

[18] M. Xie, J. Guo, X. Zhang, C. Bi, L. Zhang et al., High-efficiency pure-red Perovskite quantum-dot light-emitting diodes. Nano Lett. **22**(20), 8266–8273 (2022). 10.1021/acs.nanolett.2c03062 36251485 10.1021/acs.nanolett.2c03062

[19] M. Deng, Y. Li, X. Zhang, C. Wu, T. Zhang et al., High-efficiency pure red CsPbI_3_ QLEDs *via* strong electron affinity interface layer engineering. Adv. Opt. Mater. **12**(13), 2302758 (2024). 10.1002/adom.202302758

[20] Y.-K. Wang, F. Jia, X. Li, S. Teale, P. Xia et al., Self-assembled monolayer-based blue perovskite LEDs. Sci. Adv. **9**(36), eadh2140 (2023). 10.1126/sciadv.adh2140 37683007 10.1126/sciadv.adh2140 PMC10491221

[21] C. Zhou, J.M. Pina, T. Zhu, D.H. Parmar, H. Chang et al., Quantum dot self-assembly enables low-threshold lasing. Adv. Sci. **8**(20), 2101125 (2021). 10.1002/advs.202101125 10.1002/advs.202101125 PMC8529423 34449133

[22] X.-K. Liu, W. Xu, S. Bai, Y. Jin, J. Wang et al., Metal halide Perovskites for light-emitting diodes. Nat. Mater. **20**(1), 10–21 (2021). 10.1038/s41563-020-0784-7 32929252 10.1038/s41563-020-0784-7

[23] D.V. Talapin, J.-S. Lee, M.V. Kovalenko, E.V. Shevchenko, Prospects of colloidal nanocrystals for electronic and optoelectronic applications. Chem. Rev. **110**(1), 389–458 (2010). 10.1021/cr900137k 19958036 10.1021/cr900137k

[24] C.R. Kagan, C.B. Murray, M. Nirmal, M.G. Bawendi, Electronic energy transfer in CdSe quantum dot solids. Phys. Rev. Lett. **76**(9), 1517–1520 (1996). 10.1103/PhysRevLett.76.1517 10061743 10.1103/PhysRevLett.76.1517

[25] M.A. Boles, D. Ling, T. Hyeon, D.V. Talapin, The surface science of nanocrystals. Nat. Mater. **15**(2), 141–153 (2016). 10.1038/nmat4526 26796733 10.1038/nmat4526

[26] J. Bang, Y.Y. Sun, J.-H. Song, S.B. Zhang, Carrier-induced transient defect mechanism for non-radiative recombination in InGaN light-emitting devices. Sci. Rep. **6**, 24404 (2016). 10.1038/srep24404 27075818 10.1038/srep24404 PMC4830943

[27] K.Y. Jang, S.Y. Hwang, S.-J. Woo, E. Yoon, C.-Y. Park et al., Efficient deep-blue light-emitting diodes through decoupling of colloidal perovskite quantum dots. Adv. Mater. **36**(39), 2404856 (2024). 10.1002/adma.202404856 10.1002/adma.202404856 39109569

[28] M. Zhang, J. Hu, G. Xi, J. Tu, Q. Yang et al., Colloidal perovskite nanocrystal superlattice films with simultaneous polarized emission and orderly electric polarity *via* an *in situ* surface cross-linking reaction. ACS Nano **19**(7), 7283–7293 (2025). 10.1021/acsnano.4c17654 39932160 10.1021/acsnano.4c17654

[29] W. Wang, R. Guo, X. Xiong, H. Liu, W. Chen et al., Improved stability and efficiency of perovskite *via* a simple solid diffusion method. Mater. Today Phys. **18**, 100374 (2021). 10.1016/j.mtphys.2021.100374

[30] S. Ding, Q. Wang, W. Gu, Z. Tang, B. Zhang et al., Phase dimensions resolving of efficient and stable perovskite light-emitting diodes at high brightness. Nat. Photonics **18**(4), 363–370 (2024). 10.1038/s41566-023-01372-0

[31] J. Hu, C. Bi, K. Ren, X. Zhang, W. Wang et al., High-efficiency pure-red CsPbI_3_ quantum dot light-emitting diodes enabled by strongly electrostatic potential solvent and sequential ligand post-treatment process. Nano Lett. **24**(15), 4571–4579 (2024). 10.1021/acs.nanolett.4c00651 38565076 10.1021/acs.nanolett.4c00651

[32] Z.-L. Yan, J.-S. Benas, C.-C. Chueh, W.-C. Chen, F.-C. Liang et al., Stable blue perovskite light-emitting diodes achieved by optimization of crystal dimension through zinc bromide addition. Chem. Eng. J. **414**, 128774 (2021). 10.1016/j.cej.2021.128774

[33] Y.-Y. Zhao, Y.-F. Liu, Y.-G. Bi, C.-H. Li, Y.-F. Wang et al., Improved performance of CsPbBr_3_ light-emitting diodes based on zinc bromide passivated quantum dots. Org. Electron. **116**, 106775 (2023). 10.1016/j.orgel.2023.106775

[34] J. Pan, L.N. Quan, Y. Zhao, W. Peng, B. Murali et al., Highly efficient perovskite-quantum-dot light-emitting diodes by surface engineering. Adv. Mater. **28**(39), 8718–8725 (2016). 10.1002/adma.201600784 27529532 10.1002/adma.201600784

[35] C.B. Murray, D.J. Norris, M.G. Bawendi, Synthesis and characterization of nearly monodisperse CdE (E = sulfur, selenium, tellurium) semiconductor nanocrystallites. J. Am. Chem. Soc. **115**(19), 8706–8715 (1993). 10.1021/ja00072a025

[36] H.-H. Cho, H. Yang, D.J. Kang, B.J. Kim, Surface engineering of graphene quantum dots and their applications as efficient surfactants. ACS Appl. Mater. Interfaces **7**(16), 8615–8621 (2015). 10.1021/acsami.5b00729 25825823 10.1021/acsami.5b00729

[37] Y. Tong, E.-P. Yao, A. Manzi, E. Bladt, K. Wang et al., Spontaneous self-assembly of perovskite nanocrystals into electronically coupled supercrystals: toward filling the green gap. Adv. Mater. **30**(29), 1801117 (2018). 10.1002/adma.201801117 10.1002/adma.201801117 29870579

[38] Y. Jiang, M. Cui, S. Li, C. Sun, Y. Huang et al., Reducing the impact of Auger recombination in quasi-2D perovskite light-emitting diodes. Nat. Commun. **12**(1), 336 (2021). 10.1038/s41467-020-20555-9 33436618 10.1038/s41467-020-20555-9 PMC7804015

[39] H. Zhu, K. Miyata, Y. Fu, J. Wang, P.P. Joshi et al., Screening in crystalline liquids protects energetic carriers in hybrid perovskites. Science **353**(6306), 1409–1413 (2016). 10.1126/science.aaf9570 27708033 10.1126/science.aaf9570

[40] Z.-G. Yu, Rashba effect and carrier mobility in hybrid organic-inorganic perovskites. J. Phys. Chem. Lett. **7**(16), 3078–3083 (2016). 10.1021/acs.jpclett.6b01404 27459897 10.1021/acs.jpclett.6b01404

[41] L. Cheng, T. Jiang, Y. Cao, C. Yi, N. Wang et al., Multiple-quantum-well perovskites for high-performance light-emitting diodes. Adv. Mater. **32**(15), 1904163 (2020). 10.1002/adma.201904163 10.1002/adma.201904163 31592566

[42] J. Shamsi, A.S. Urban, M. Imran, L. De Trizio, L. Manna, Metal halide perovskite nanocrystals: synthesis, post-synthesis modifications, and their optical properties. Chem. Rev. **119**(5), 3296–3348 (2019). 10.1021/acs.chemrev.8b00644 30758194 10.1021/acs.chemrev.8b00644 PMC6418875

[43] M. Zhang, C. Bi, Y. Xia, X. Sun, X. Wang et al., Water-driven synthesis of deep-blue perovskite colloidal quantum wells for electroluminescent devices. Angew. Chem. Int. Ed. **62**(12), e202300149 (2023). 10.1002/anie.202300149 10.1002/anie.202300149 36692366

[44] K. Chen, D. Zhang, Q. Du, W. Hong, Y. Liang et al., Synergistic halide- and ligand-exchanges of all-inorganic perovskite nanocrystals for near-unity and spectrally stable red emission. Nanomaterials **13**(16), 2337 (2023). 10.3390/nano13162337 37630921 10.3390/nano13162337 PMC10458086

[45] J. Zhang, T. Zhang, Z. Ma, F. Yuan, X. Zhou et al., A multifunctional “halide-equivalent” anion enabling efficient CsPb(Br/I)_3_ nanocrystals pure-red light-emitting diodes with external quantum efficiency exceeding 23%. Adv. Mater. **35**(8), 2209002 (2023). 10.1002/adma.202209002 10.1002/adma.202209002 36493461

[46] P. Zeng, X. Ren, L. Wei, H. Zhao, X. Liu et al., Control of hot carrier relaxation in CsPbBr_3_ nanocrystals using damping ligands. Angew. Chem. **134**(15), e202111443 (2022). 10.1002/ange.202111443 10.1002/anie.202111443 34997699

[47] J. Yin, P. Maity, R. Naphade, B. Cheng, J.-H. He et al., Tuning hot carrier cooling dynamics by dielectric confinement in two-dimensional hybrid perovskite crystals. ACS Nano **13**(11), 12621–12629 (2019). 10.1021/acsnano.9b04085 31613089 10.1021/acsnano.9b04085

[48] K. Fan, H. Liu, T. Pan, Y. Wu, Y. Hai et al., Engineering carrier thermalization, relaxation, and funneling in mixed 3D/quasi-2D CsPbI_3_ perovskite nanocrystals. ACS Nano **19**(30), 27552–27562 (2025). 10.1021/acsnano.5c06756 40704411 10.1021/acsnano.5c06756

