# Supplementary material for: Exceeding 30% Efficiency of Red Perovskite Quantum Dot Light-Emitting Diodes via Interparticle Energy Dissipation Suppression
Source: Nanomicro Lett. 2026 Mar 19;18:294. doi: 10.1007/s40820-026-02156-1 (PMC13003073; doi:10.1007/s40820-026-02156-1)
Supplement: Supplementary file 1 — Supplementary file1 (DOCX 6534 KB) [file 40820_2026_2156_MOESM1_ESM.docx]

# Exceeding 30% Efficiency of Red Perovskite Quantum Dot Light-Emitting Diodes via Interparticle Energy Dissipation Suppression

Zhiwei Yao^†1,3,4^, Changsheng Liang^†1,4^, Chenghao Bi^2^*, Wenyuan Zhou^1,4^, Ke Ren^2^, Ming Deng^1,4^, Shuo Ding^1,4^*, Chaoyu Xiang^1,3,4^*

1. Laboratory of Advanced Nano-Optoelectronic Materials and Devices, Ningbo Institute of Materials Technology and Engineering, Chinese Academy of Science, Ningbo, Zhejiang, 315201, China.
2. College of Physics and Optoelectronic Engineering, Harbin Engineering University, Harbin 150001, China.
3. University of Chinese Academy of Sciences, No.1 Yanqihu East Road, Huairou District, Beijing, China.
4. Laboratory of Advanced Nano-Optoelectronic Materials and Devices, Qianwan Institute of CNITECH, Ningbo, P. R. China, Ningbo 315300, China.

Email: [chenghao.bi@hrbeu.edu.cn](mailto:chenghao.bi@hrbeu.edu.cn); [dingshuo@nimte.ac.cn](mailto:dingshuo@nimte.ac.cn); [xiangchaoyu@nimte.ac.cn](mailto:xiangchaoyu@nimte.ac.cn)

***Supplementary Figures and Tables***


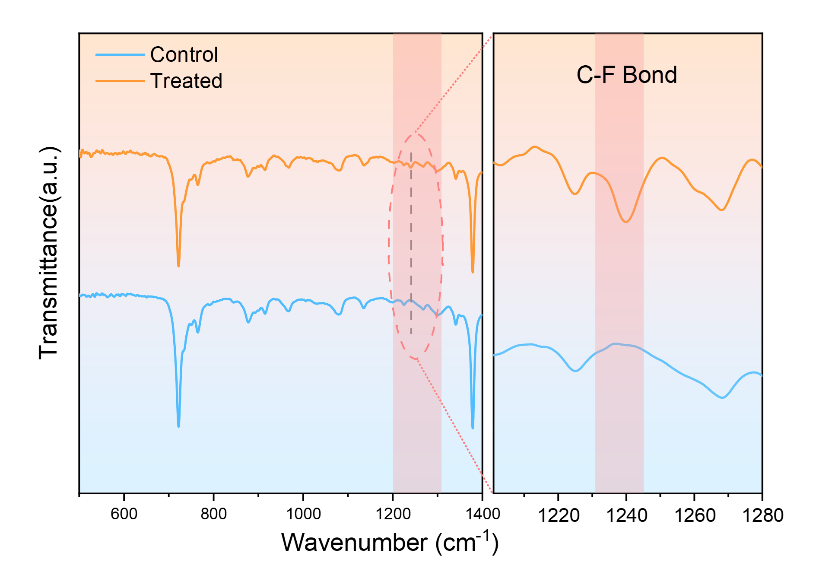


Figure S1. FT-IR spectra of the control and 11-PFHA-treated QDs


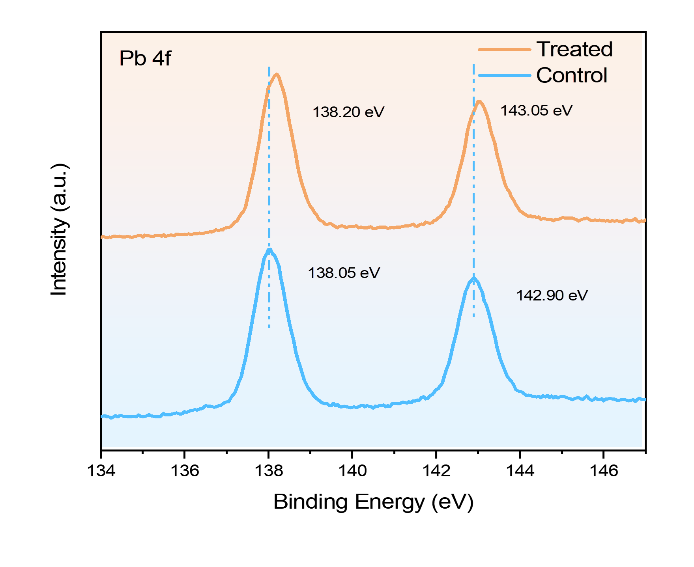


Figure S2. XPS results of the control and 11-PFHA-treated QDs


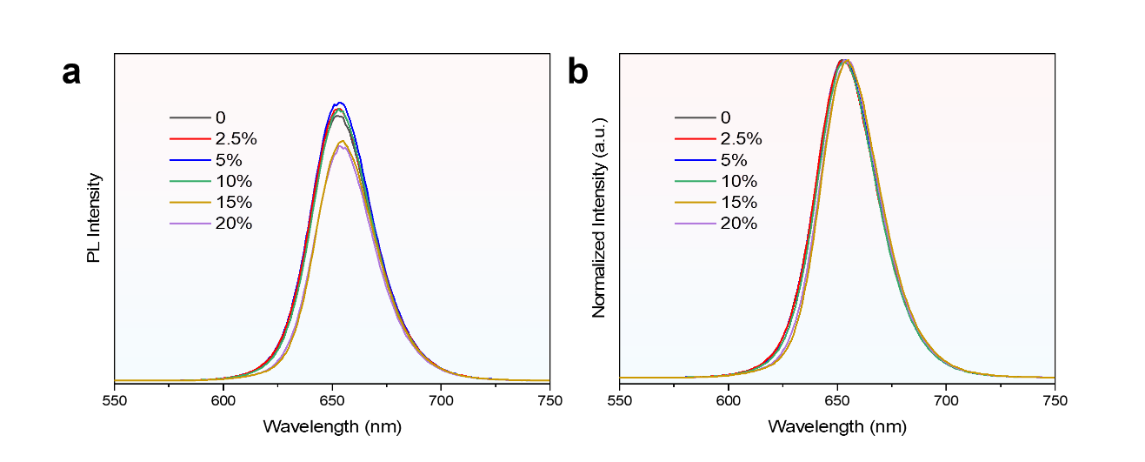


**Figure S3. a** The PL spectra and **b** the normalized PL spectra of QDs treated with different dose of 11-PHFA


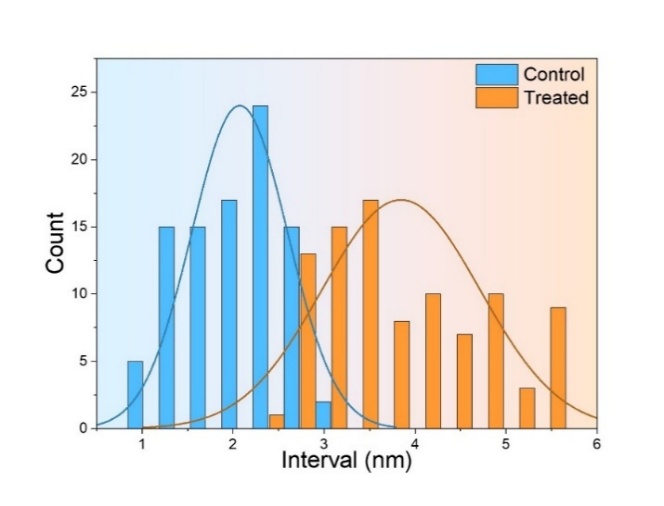


Figure S4. The distribution of the interval of control and treated QDs in TEM measurements


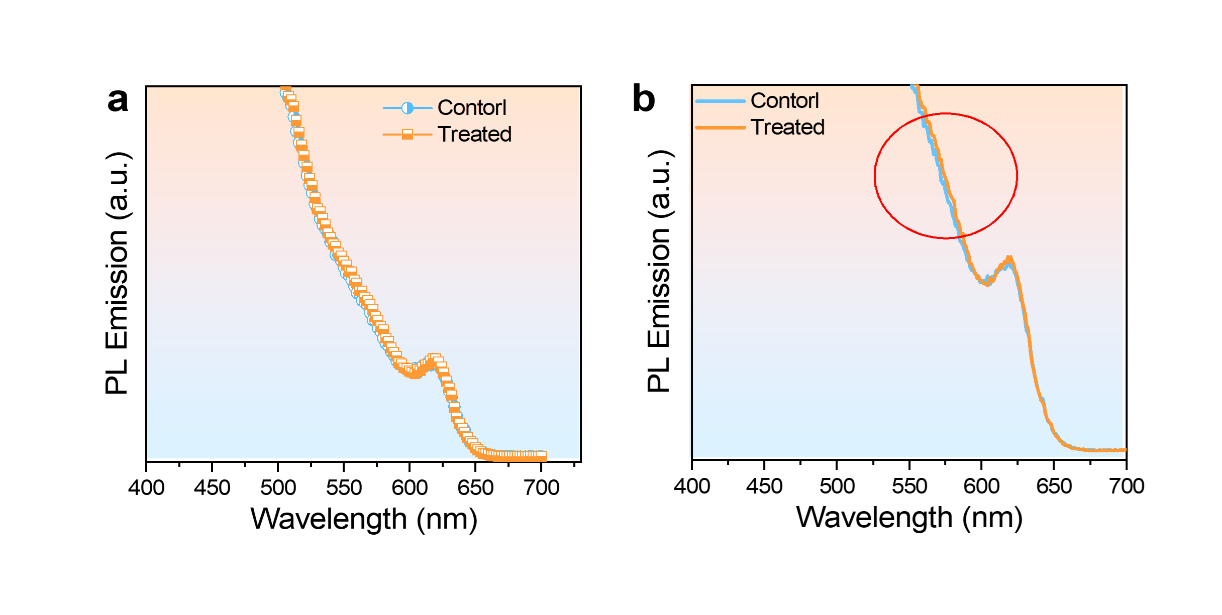


Figure S5. The a UV spectra and b PLE spectra of the control and treated QDs


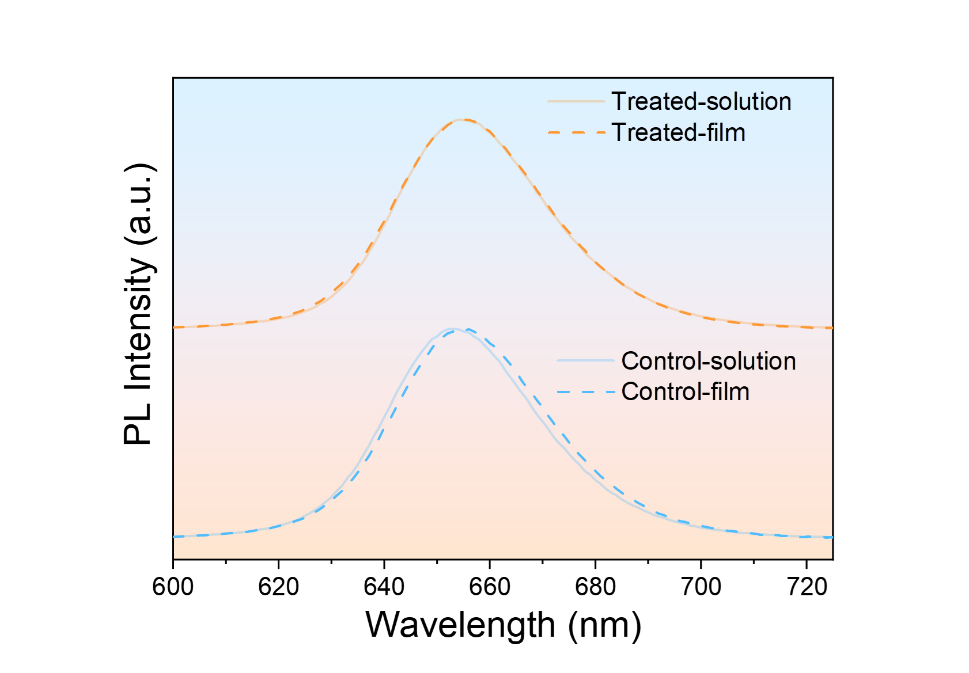


Figure S6. PL emission spectra of control and treated QDs in solution and film (657 nm)


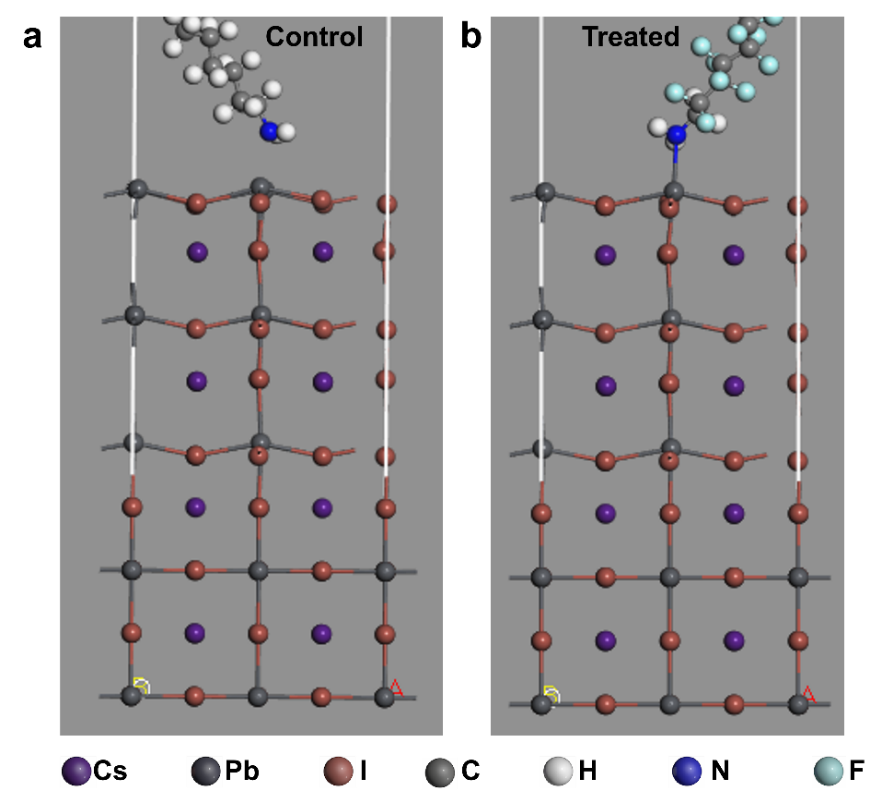


Figure S7. The structural diagrams of QDs passivated by a alkylamine and b 11-PFHA


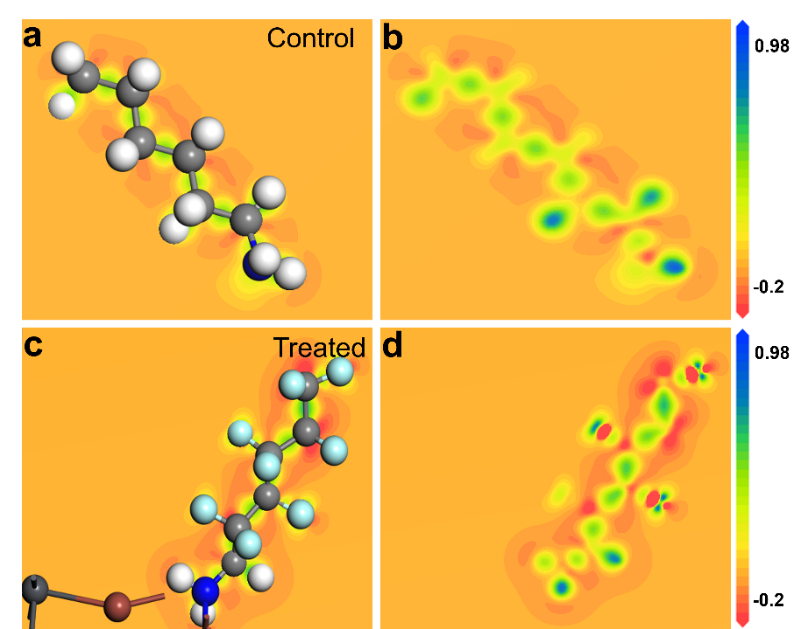


Figure S8. Schematic diagram of (the cross-section along the ligand molecule) electron density difference between a, b alkylamine and c, d 11-PFHA (the cross-section perpendicular to the one shown in Figure S3)


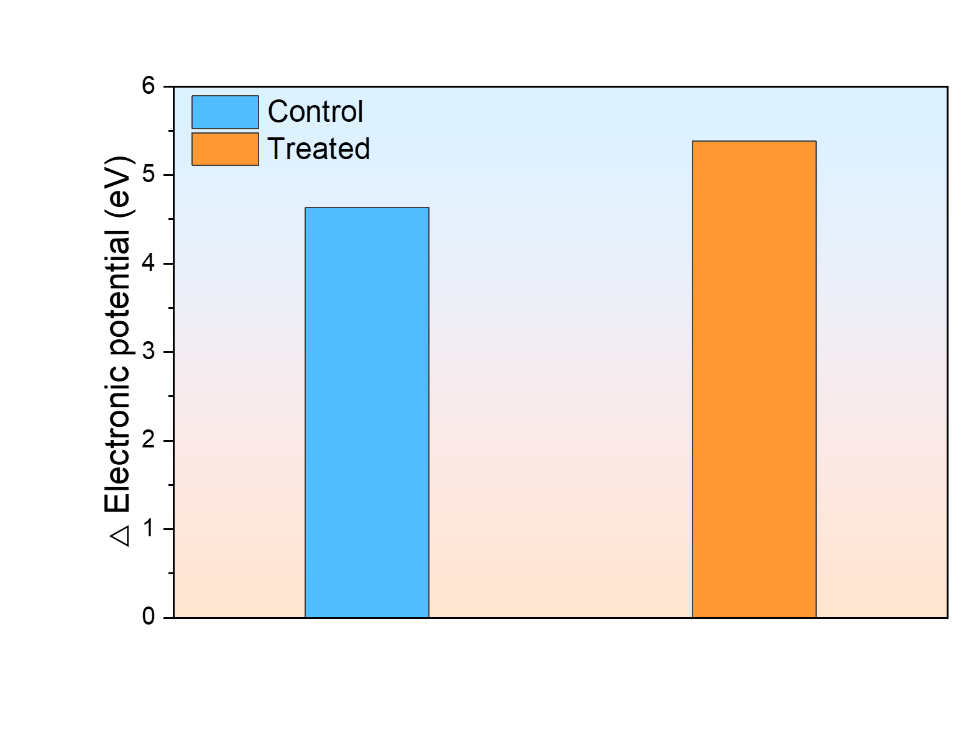


Figure S9. The electron potential barriers in QDs passivated with different ligands


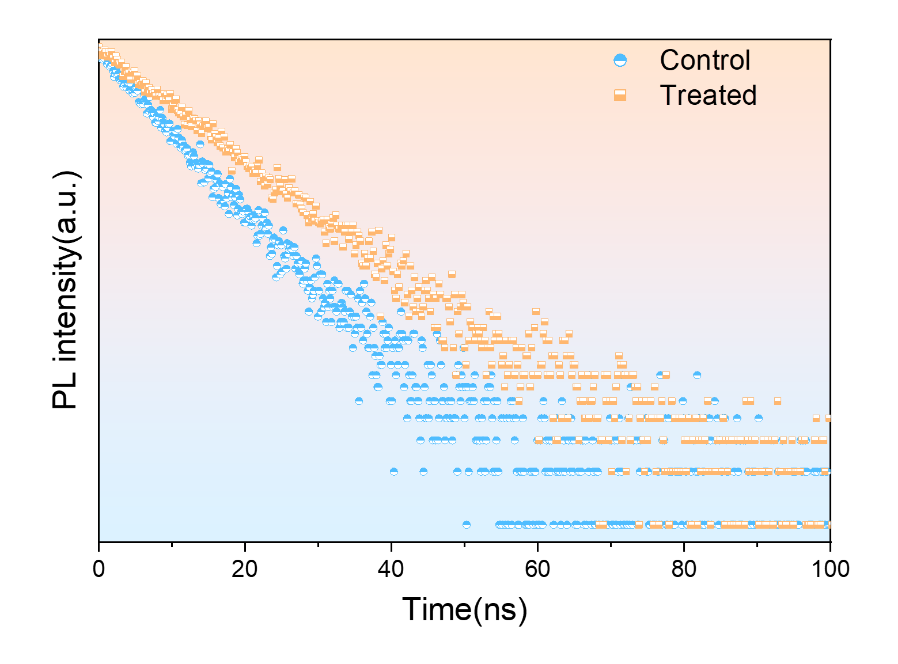


Figure S10. The PL Decay measurements of the control and treated QDs


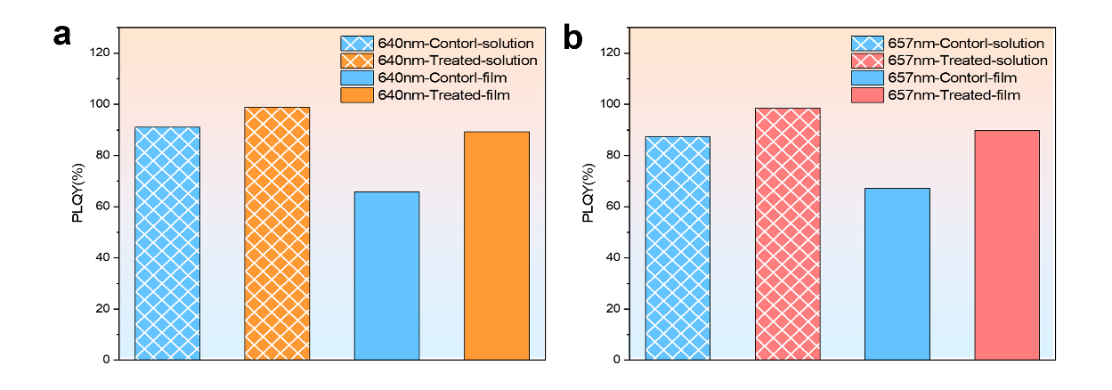


Figure S11. PLQY results of the control and treated QDs emission at a 640 nm and b 657 nm in solution and film state


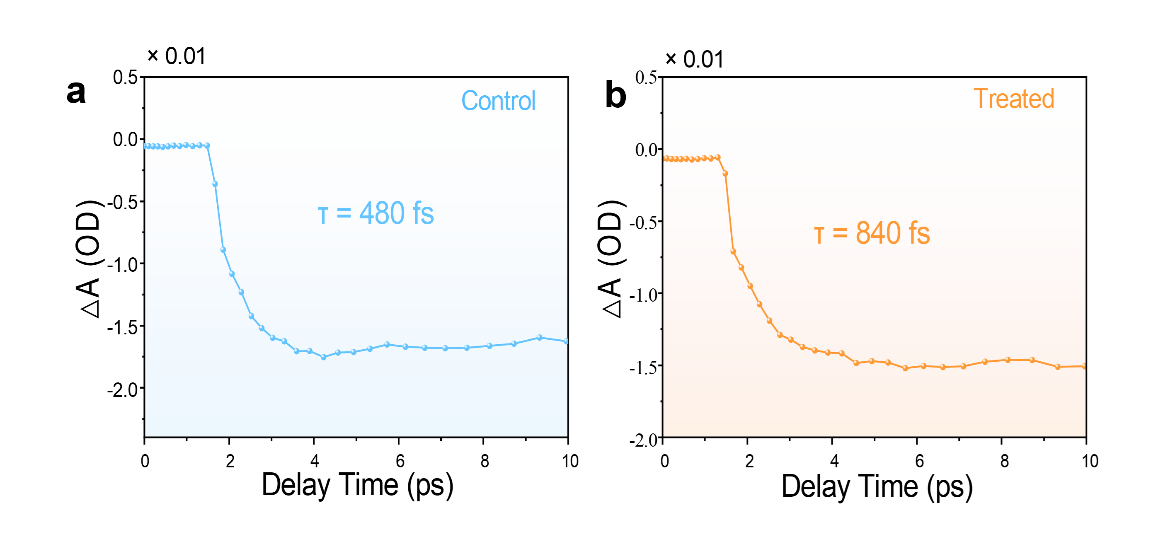


Figure S12. TA spectra of GSB peaks establishment dynamics process of a control and b treated QDs


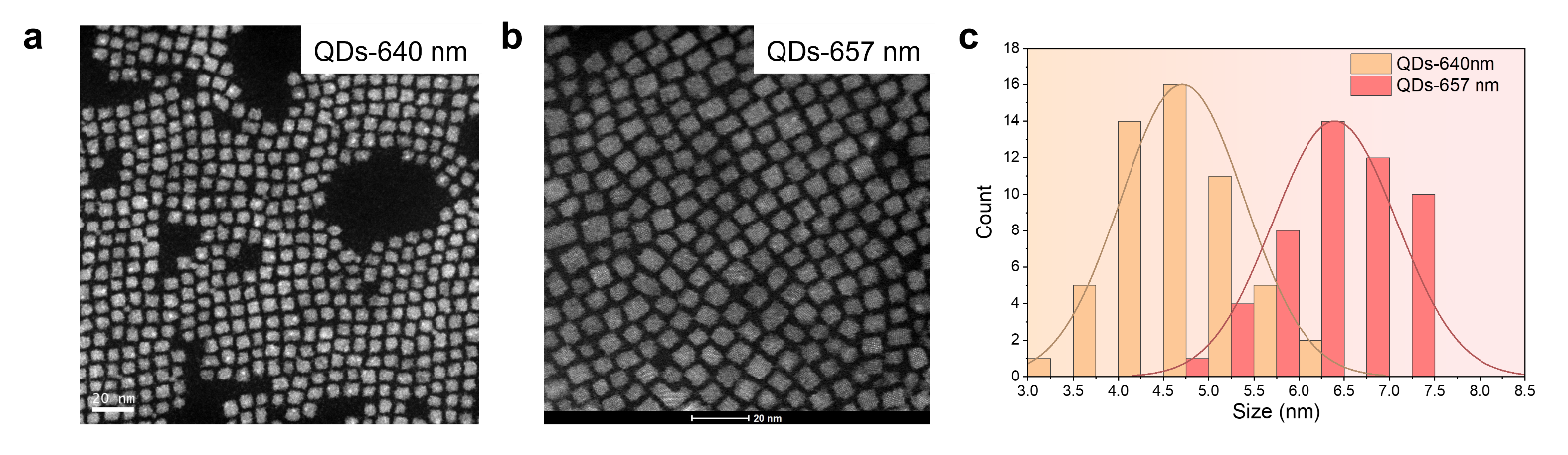


Figure S13. The TEM pictures of QDs emission at a 640nm and b 657nm. c The statistic of the size of QDs emission at 640nm and 657nm


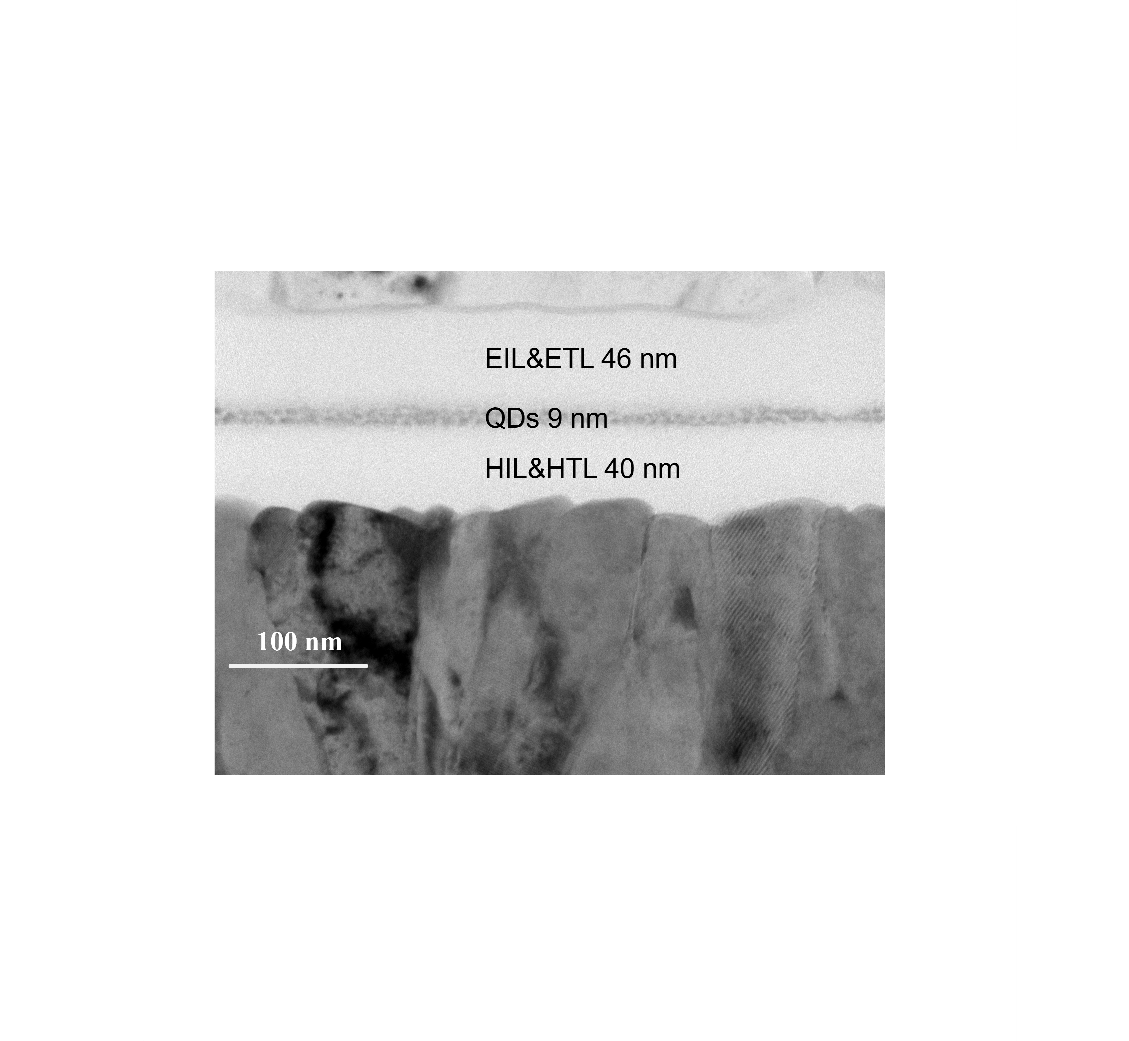


Figure S14. Cross-sectional TEM images of LEDs based on CsPbI_3_ QD emission at 640nm


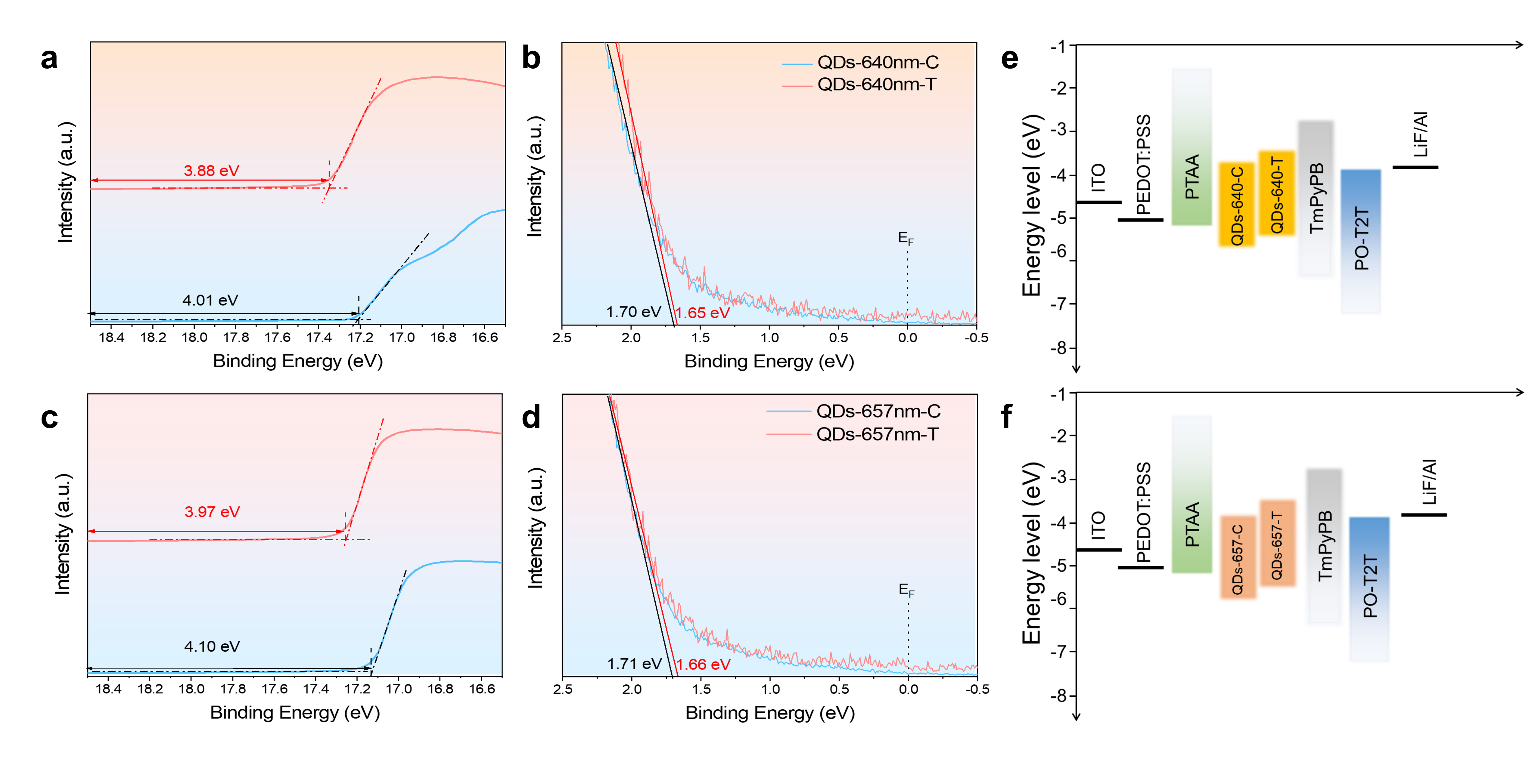


Figure S15. The UPS details of the control and treated QDs a, b emission at 640 nm and c, d 657 nm. The energy landscape of the QD emission at e 640 nm and f 657 nm


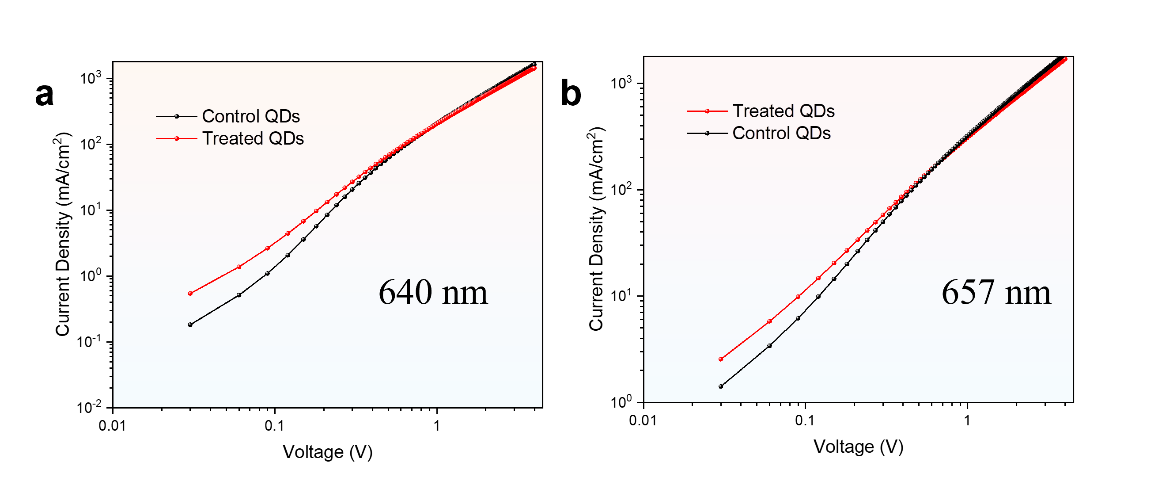


**Figure S16.** The current density vs voltage curves of the hole only devices based on control QDs and 11-PFHA QDs emission at **a** 640 nm and **b** 657 nm


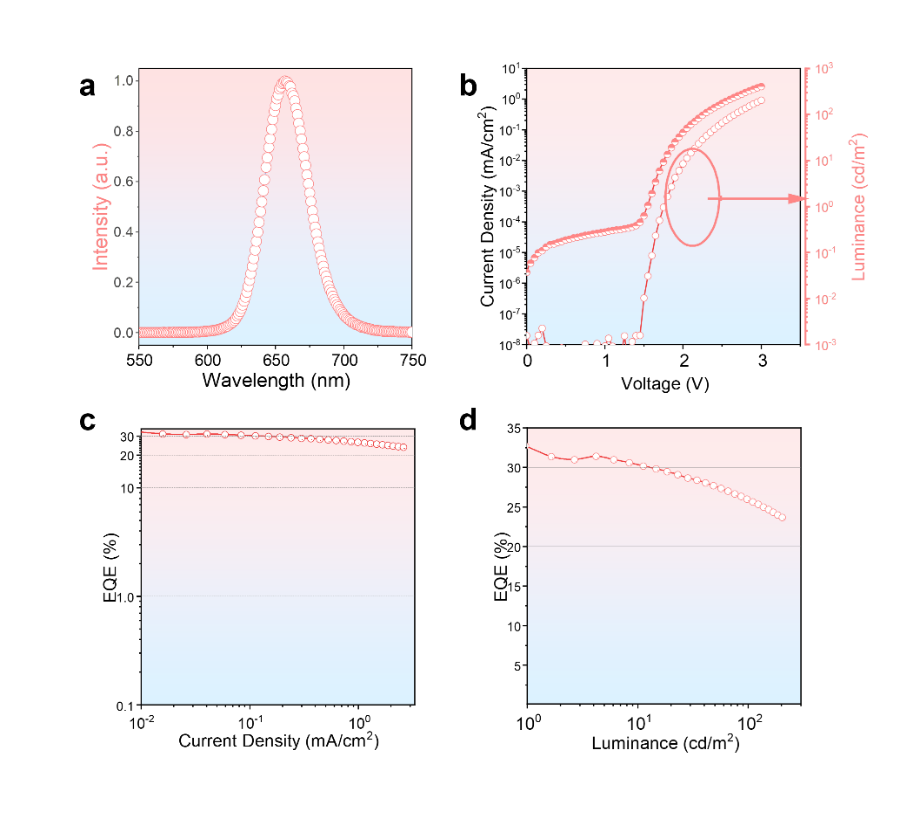


Figure S17. a EL spectra b Current density vs Voltage vs Luminance curves c EQE vs Current density curve and d EQE vs Luminance curve of 11-PFHA treated QDs measured by the group of Zhang Xiaoyu in Jilin University


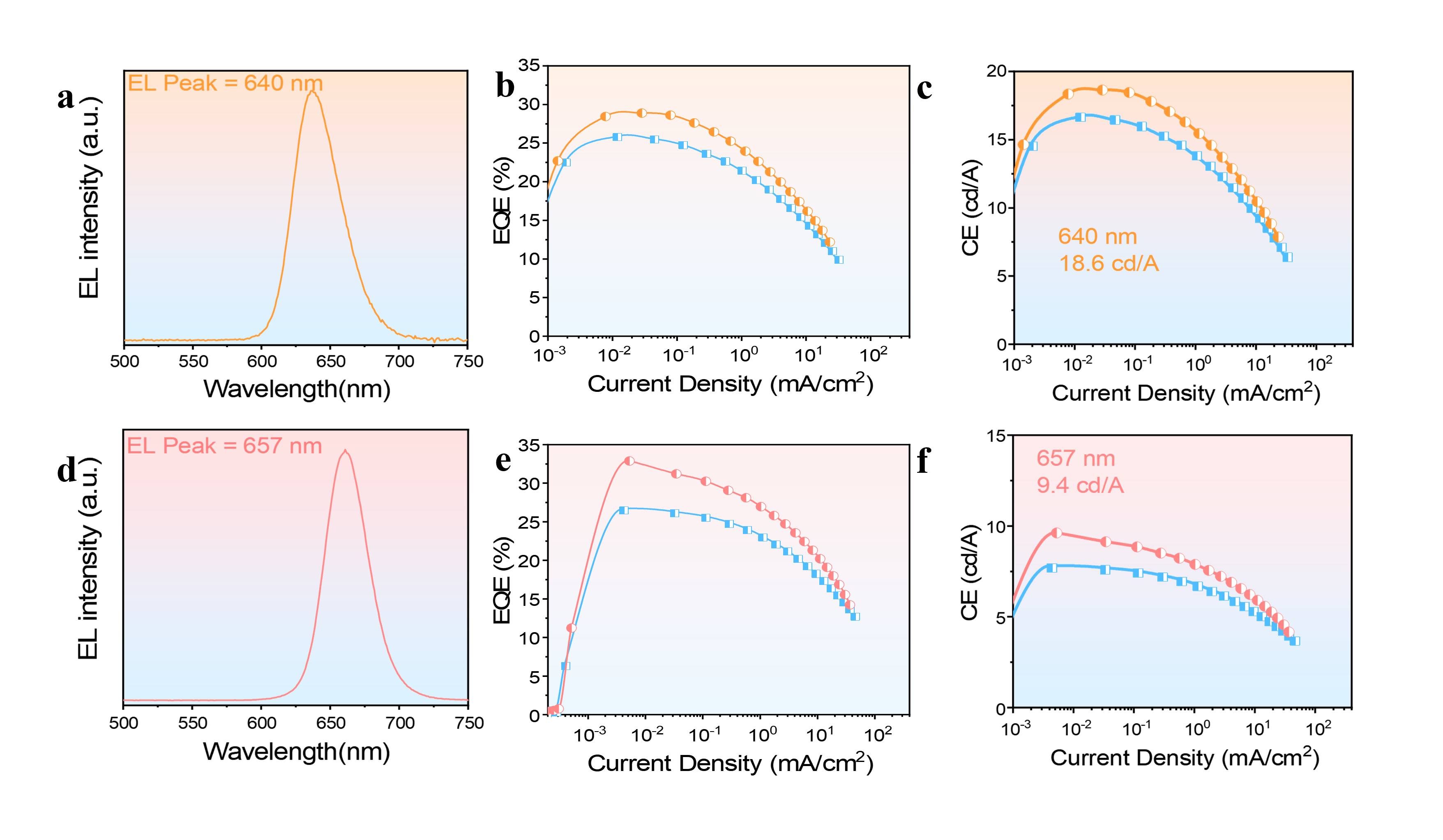


**Figure S18.** **a** The EL spectra **b** EQE vs current density and **c** CE vs current density curve of PeLEDs based on QDs emission at 640nm. **d** The EL spectra **e** EQE vs current density and **f** CE vs current density curve of PeLEDs based on QDs emission at 657nm


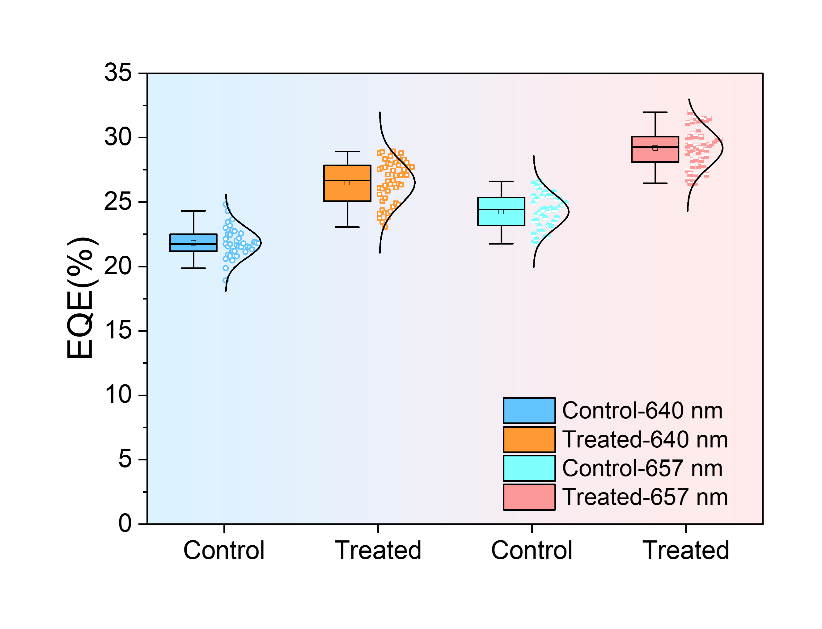


**Figure S19.** The distribution of the peak EQE of the PeLEDs fabricated with control and treated QDs emission at 640nm and 657nm


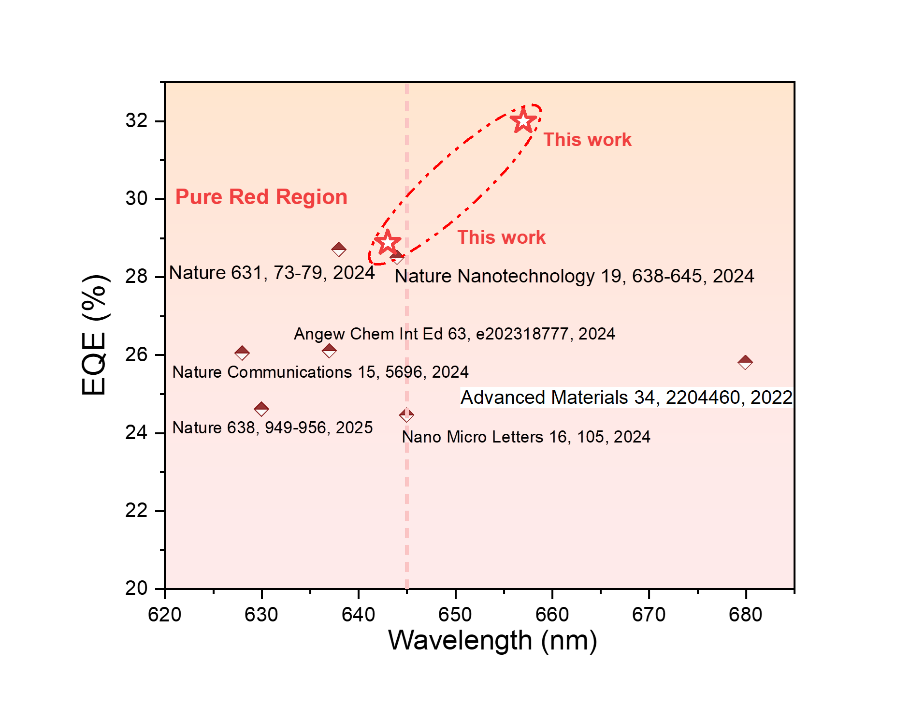


Figure S20. Comparison of the EQE of devices based on CsPbI_3_ in our work with others available from the current literature


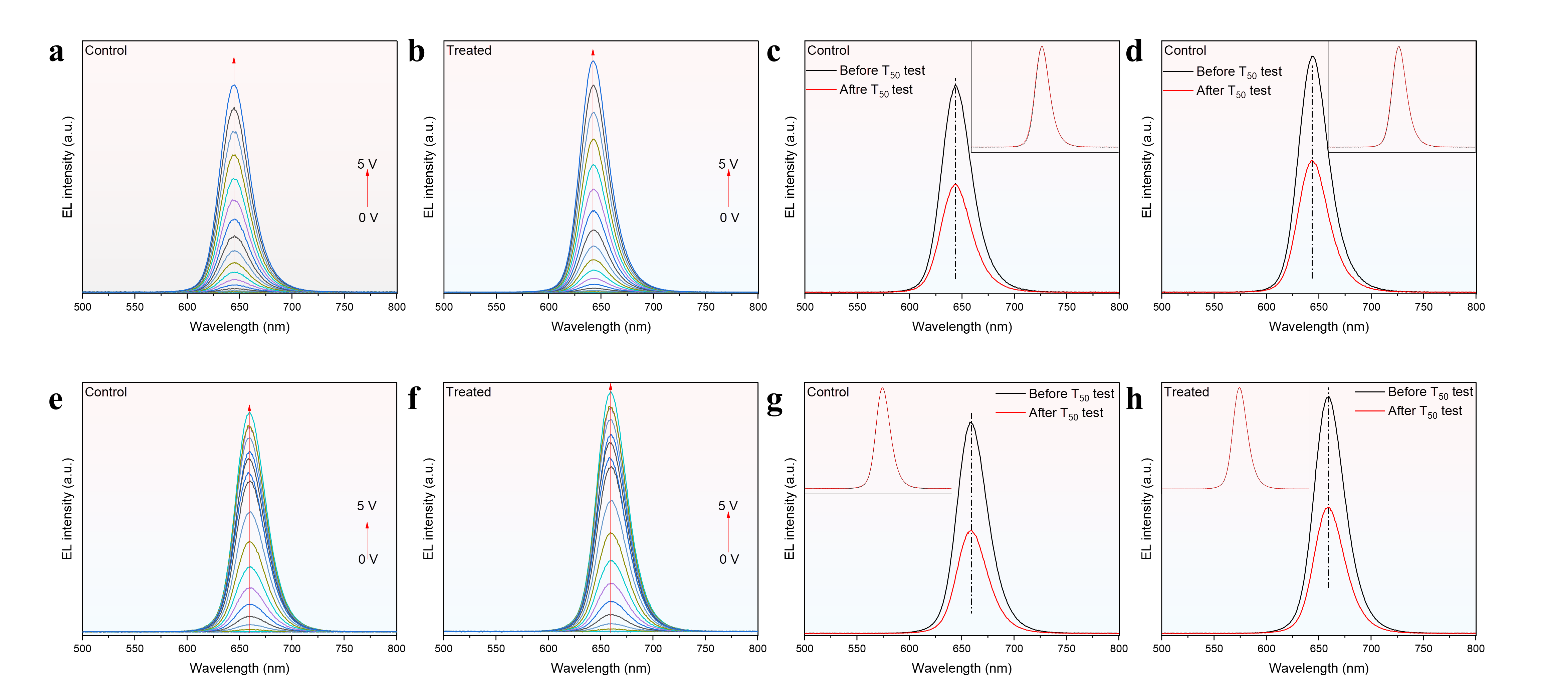


**Figure S21.** The PL spectra at different bias of devices based on **a**, **e** control QDs and **b, f** 11-PFHA treated QDs. The PL spectra before and after T_50_ tests of devices based on the **c, d** control and **g, h** 11-PFHA treated QDs, the normalized EL spectra are embedded in the corresponding EL spectra


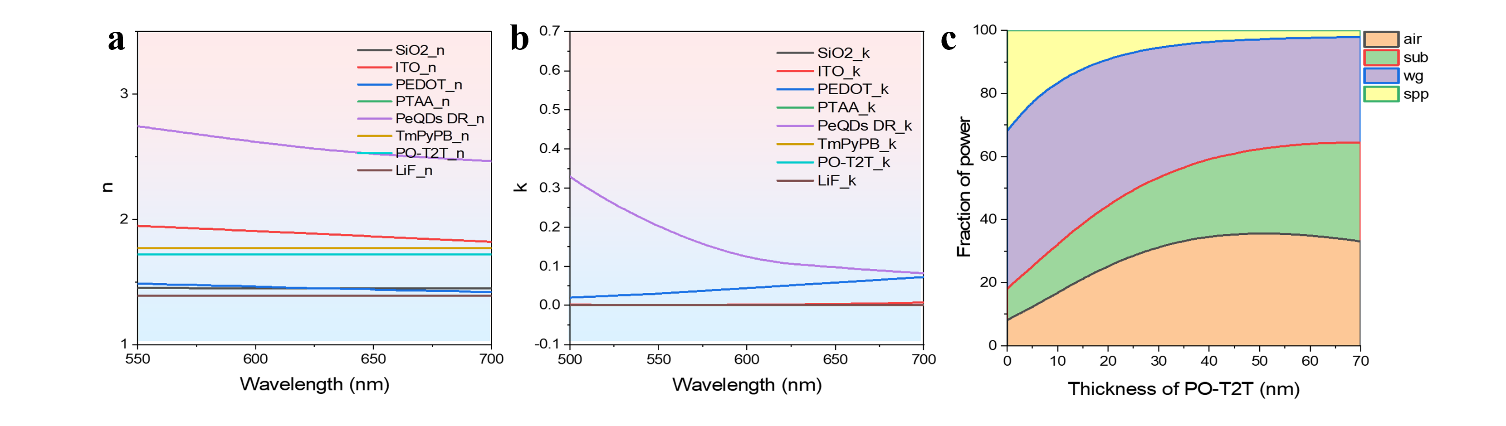


**Figure S22.** **a** Refractive index n and **b** extinction coefficient k of each function layer. **c** Relative ratios of each mode in 657 nm-PeLED without versus ETL (PO-T2T) thickness

As shown in the profile, the light extraction efficiency of our device is 35.5% when the thickness of PO-T2T&TmPyPB is 45nm, which is exactly the thickness of our devices shown in **Figure S12**


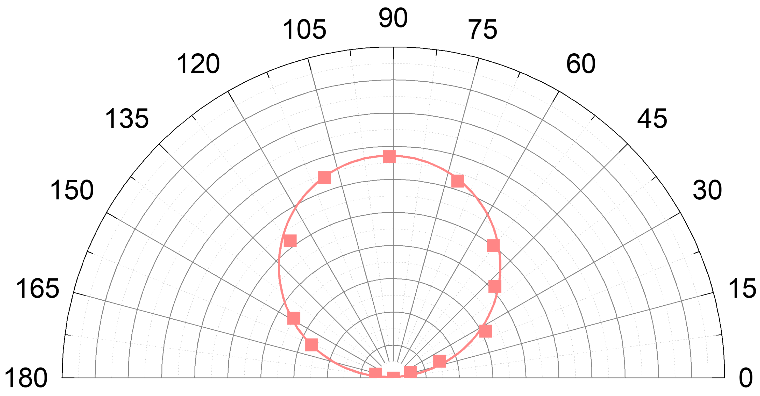


**Figure S23.** Angular dependent electroluminescence (EL) intensity of red PeLED


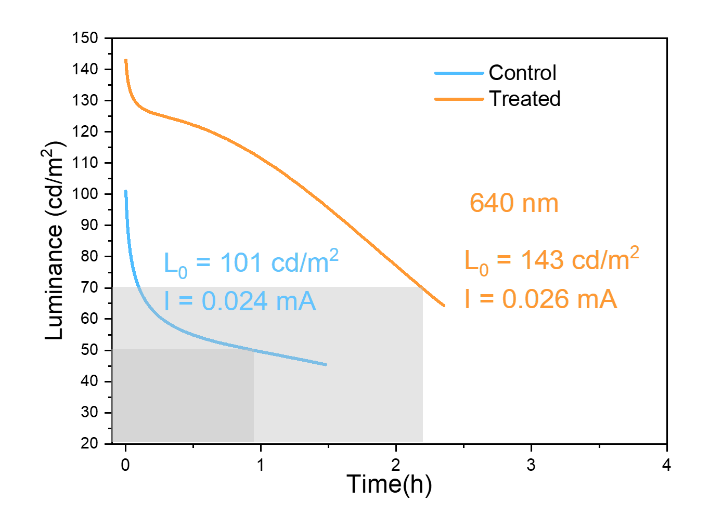


Figure S24. The operational lifetime of PeLEDs based on control QDs and treated QDs (640 nm)


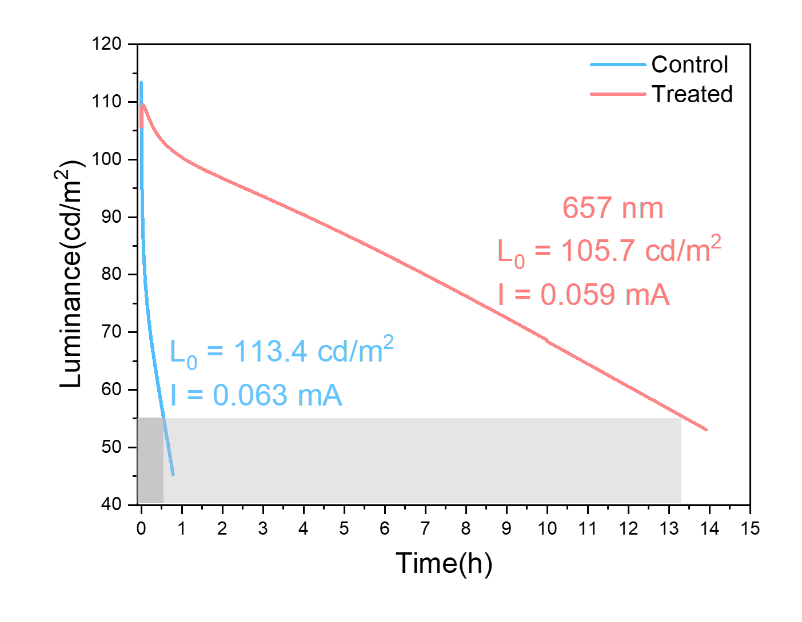


Figure S25. The operational lifetime of PeLEDs based on control QDs and treated QDs (657 nm)

**Table S1.** Fitting data of the PL Decay measurement.

| Sample | A_1_(%) | τ_1_(ns) | A_2_(%) | τ_2_ (ns) | τ_ave_ (ns) |
| --- | --- | --- | --- | --- | --- |
| Control | 100 | 10.15 | - | - | 10.15 |
| Treated | 100 | 13.62 | - | - | 13.62 |
